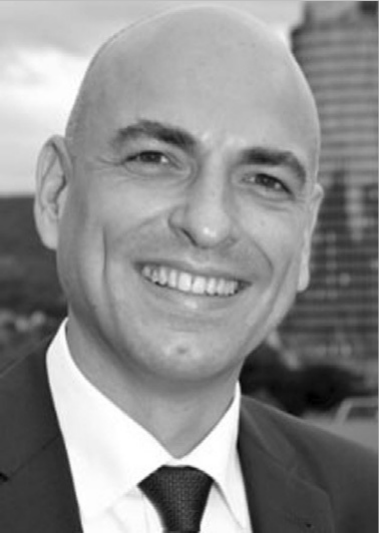# Sustainable Energy Storage: Recent Trends and Developments toward Fully Organic Batteries

**DOI:** 10.1002/cssc.201901545

**Published:** 2019-08-22

**Authors:** Christian Friebe, Alexandra Lex‐Balducci, Ulrich S. Schubert

**Affiliations:** ^1^ Laboratory of Organic and Macromolecular Chemistry (IOMC) Friedrich Schiller University Jena Humboldtstraße 10 07743 Jena Germany; ^2^ Center for Energy and Environmental Chemistry Jena (CEEC Jena) Friedrich Schiller University Jena Philosophenweg 7a 07743 Jena Germany

**Keywords:** electrochemistry, energy storage, hybrid metal–organic batteries, organic batteries, redox chemistry

## Abstract

In times of spreading mobile devices, organic batteries represent a promising approach to replace the well‐established lithium‐ion technology to fulfill the growing demand for small, flexible, safe, as well as sustainable energy storage solutions. In the last years, large efforts have been made regarding the investigation and development of batteries that use organic active materials since they feature superior properties compared to metal‐based, in particular lithium‐based, energy‐storage systems in terms of flexibility and safety as well as with regard to resource availability and disposal. This Review compiles an overview over the most recent studies on the topic. It focuses on the different types of applied active materials, covering both known systems that are optimized and novel structures that aim at being established.

## Graphical abstract

**The search for the green battery** is at the center of numerous efforts during the last years. In particular, the replacement of environmentally questionable metals by more sustainable organic materials is on the current research agenda. This review presents recent results regarding the developments of organic active materials for electrochemical energy storage.

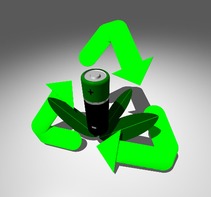

## 1 Introduction

**Figure 1:**
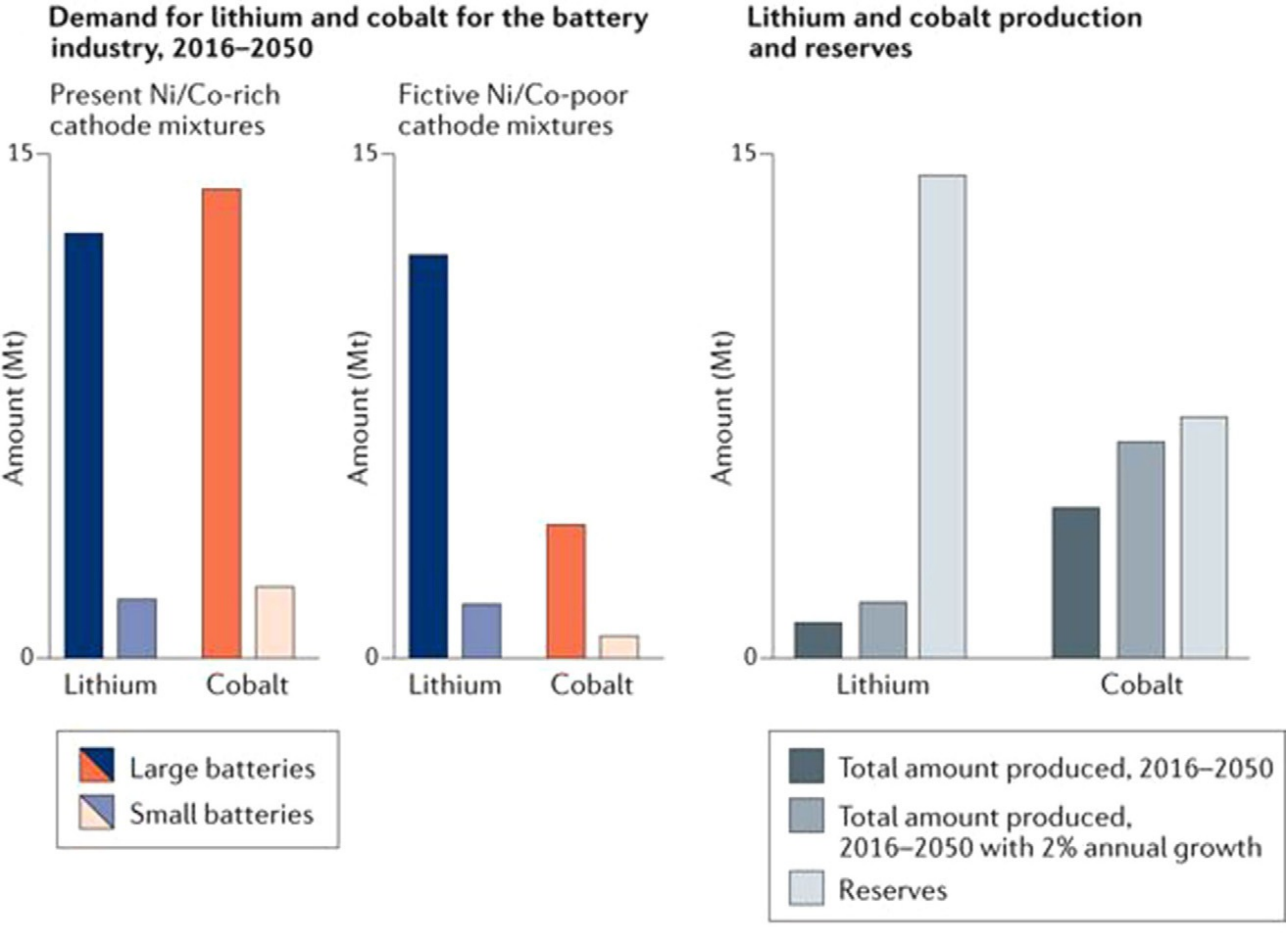
Predicted demands (left), production, and reserves (right) of lithium and cobalt resources for the battery industry (reprinted by permission from Springer Nature, Copyright 2018).^2^

Nowadays, a high and steadily increasing demand for technologies and possibilities for the storage of electrical energy exists not only within the industrial world but also in the developing countries. A particular, ever‐growing interest in small, lightweight, mechanically flexible and stable, safe, as well as inexpensive energy storage is present due to quickly emerging mobile devices, smart packaging and clothing, as well as the rising Internet of Things. However, the current leader in mobile energy storage, the lithium‐ion battery, exhibits several disadvantages with regard to the stated requirements. In particular, safety issues render such batteries unsuitable for applications that include large mechanical stress, potentially even leading to leakage or breaking of the battery, which would be disastrous in case of lithium‐based batteries.^1^ Furthermore, the current lithium‐ion technology depends on the provision with large amounts of critical resources. Already today, half of the global production of lithium is used for batteries, which will definitely increase further in the future. Another aspect that has to be taken into consideration is that most of the known reserves are located in politically or climatically problematic regions of the world.^2^ But current lithium‐ion batteries do not only contain lithium but also large amounts of nickel, manganese, and cobalt. The latter exhibits particularly large restrictions since over 60 % of today's production and of the known reserves are located in politically instable regions and can barely match the predicted demands for the next 30 years (Figure 1); nickel does not perform much more promising.^2^ Finally, the disposal of current lithium‐ion batteries and the recycling of the contained materials is still an issue under investigation.^3^ Consequently, alternative active materials have to be found to enable sustainable electrochemical energy storage.^4^

Organic batteries, which utilize organic or polymeric active materials instead of metals or metal oxides, represent the most promising approach to overcome the technical and economical restrictions of the established metal‐based systems. They do not rely on controversial metal deposits, but the active materials can prospectively be synthesized from renewable resources in the future.^5^ Furthermore, they provide a superior processability, enabling the use of printing techniques (e.g., screen printing, inkjet printing) and various other casting methods (e.g., doctor blading) as well as roll‐to‐roll manufacturing and allow the construction of mechanically flexible devices.^6^

Based on the discovery of the conductivity of conjugated polymers in 1977,^7^ organic batteries were firstly developed already in the 1980s^8^ and commercialized within a few years by Bridgestone/Seiko and VARTA/BASF.^9^ However, those systems were based on poly(pyrrole) and poly(aniline), which did not provide a stable working voltage and were consequently taken off the market. Not before 2002, Nakahara et al. presented the next step in this research field. They reported a working battery that was based on the 2,2,6,6‐tetramethyl‐4‐piperidinyl‐*N*‐oxyl (TEMPO) radical and started a new and much larger wave of new materials and concepts toward the development of organic batteries.^10^ Since then, numerous organic active materials intended for the utilization in batteries were investigated.^11^ This Review gives a comprehensive and critical overview over the systems that were developed recently since our last survey in 2016^12^ with a focus on all‐organic approaches.

## 2 Background

### 2.1 Working principle

**Scheme 1:**
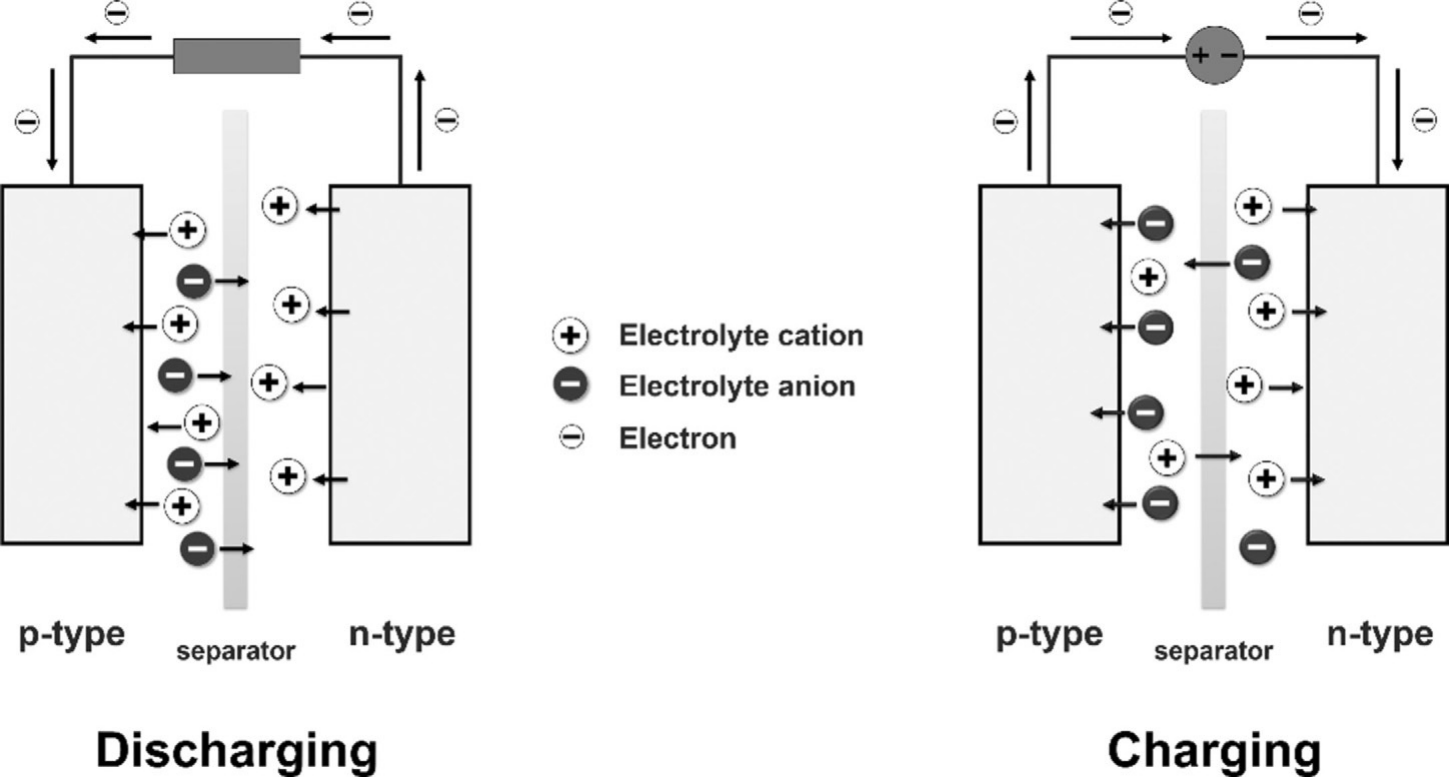
Discharging and charging process of an electrochemical cell (adapted with permission from Springer Nature, Copyright 2017).^13^

Batteries are based on the concept of an electrochemical cell, that is, two electrodes made of redox‐active materials that are placed in an electrolyte, with a separator (e.g., a salt bridge, a semipermeable membrane) between them, and connected electrically. Electrons move from the material with the lower redox potential (the negative pole) to the material with the higher redox potential (the positive pole) until the former is completely oxidized and the latter is completely reduced—the cell is discharged. When an external force, namely an electric current in the opposite direction, is applied, the process is reversed, and the cell is charged. The active materials are therefore classified into one of three categories: n‐type materials (“negative”; low redox potential), p‐type materials (“positive”; high redox potential), and b‐type materials (“bipolar”; medium or high and low redox potential).11c During the discharging and charging process, ions move through the electrodes and the electrolyte to allow charge neutrality (Scheme 1).

In this Review, an organic battery is a battery that possesses at least one electrode with an organic redox‐active material. If both electrodes are based on organic active materials, it is called an all‐organic or fully organic battery (although the electrolyte can contain inorganic ions). However, since metal electrodes usually provide established and well‐known redox processes, most of the novel organic materials are investigated in metal–organic hybrid cells with one electrode based on a metal (e.g., lithium, sodium, potassium, magnesium) and an electrolyte containing respective metal cations. This allows the execution of basic studies in a more reliable way. Usually the metal constitutes the negative pole since most applied organic materials feature redox potentials that lie above those of the commonly used metals. Nevertheless, an all‐organic battery without metallic active materials is eventually desired.

### 2.2 Performance parameters

To assess the suitability of an active material or a whole cell setup, several performance parameters are theoretically and experimentally accessible:^14^

The cell voltage (*V* [V]) is the working voltage of a cell. It is determined from the voltage profile of a charge/discharge experiment, which shows a plateau in an ideal case, allowing an easy reading of the voltage. In real cases, however, the voltage can change during the course of charging or discharging (a “sloping” voltage), which necessitates the determination of an arithmetic average. The voltages measured during discharging and charging usually differ; their ratio is stated as the voltage efficiency (*η*_V_ [%]). The maximum voltage of a cell is called the theoretical voltage (*V*_theo_ [V]), which can be calculated from the redox potentials (*E*) of the employed active materials according to *V*_theo_=*E*_cathode_−*E*_anode_.

The capacity (*C* [Ah]) describes how much charge can be stored in a cell. It depends mostly on the number of redox centers, the number of transferred electrons per redox center, and the accessibility of the redox centers. The maximum, resulting from the first two values, is represented by the theoretical capacity (*C*_theo_ [Ah]). Another important parameter is the specific capacity (*C*_spec_ [mAh g^−1^]), which is the amount of storable charge referred to the mass of applied active material. It is crucial in particular for mobile applications and can be determined via *C*_spec_=$\frac{n\cdot F}{M}$ (*n* … number of transferred electrons per redox reaction, *F* … Faraday constant, *M* … molar mass of the redox‐active unit). Usually, the limiting capacity is stated. For the whole cell, *C*_spec_ is calculated according to *C*_spec, cell_^−1^=*C*_spec, anode_^−1^+*C*_spec, cathode_^−1^. The capacities measured during discharging and charging often differ from each other due to reversible and irreversible losses. The ratio between discharge and charge capacity states an important value, which is called the coulombic efficiency (*η*_c_ [%]).

The energy (*E* [Wh]) that can be stored in a cell can be calculated from the determined cell voltage and capacity (*E*=*V*⋅*C*). Accordingly, the maximum theoretical energy (*E*_theo_ [Wh]) and the specific energy (*E*_spec_ [mWh g^−1^]) are calculated using the theoretical and specific capacity, respectively. The energy density (*E*_dens_ [Wh L^−1^]) is determined by the storable energy with respect to the volume of the material. The ratio between discharge and charge energy is the energy efficiency (*η*_W_ [%]), which is another important parameter for the evaluation of the cell performance.

The performance of a cell at different charge/discharge currents is expressed by the rate capability, which is normally described by the highest current density that gives a capacity similar to the capacities at significantly lower current densities. The current density is usually given in terms of the C‐rate, which is derived from the current (*i*) that is required to charge the cell within 1 h (C‐rate=$\frac{i{}_{\mathrm{applied}}}{i{}_{1\;h}}$ ).

In Table 1, the latest state‐of‐the‐art values for lithium‐ion^15^ and organic^12^ batteries are stated for comparison.

**Table 1:** Latest benchmark values for lithium‐ion and organic batteries.^12^, ^15^ Specific capacities and energies are stated for anode and cathode as a combined system.

Battery system	*V* [V]	*C*_spec_ [mAh g^−1^]	*E*_spec_ [mWh g^−1^]	Rate capability	Cycle stability
lithium ion	≤4.5	≤200	≤650	≤5 C	1000 to 2000
hybrid organic	≤4.0	≤200^[a]^	≤400^[a]^	≤20 C	≤1500
fully organic	≤1.3	≤50	≤90	≤320 C	≤250

[a] With respect to a lithium metal anode.

### 2.3 Electrode components

Since organic active materials generally do not possess sufficient electrical conductivity, conductive agents have to be added during electrode preparation to ensure charge transport between the current collector and the redox‐active species during the discharging and charging process. Beside a high electronic conductivity, the conductive agent has also to provide a large surface area to ensure an intimate contact to the active material. Furthermore, since ion transport into and out of the electrode is crucial for unhindered discharging and charging in terms of charge neutrality, the conductive additive must offer a kind of porous and flexible network to allow penetration of the electrolyte and ion migration as well as compensation of volume changes, which occur due to ion insertion/release. The used conductive additives are usually based on nanostructured carbon materials, for example, carbon nanoparticles,^16^ mesoporous carbon,^17^ vapor‐grown carbon fibers,^18^ graphene,^19^ and carbon nanotubes.^20^ For an optimized interaction, the active material has to be thoroughly mixed with the conductive additive to form a composite electrode. Alternatively, several systems were recently developed that include a more intimate conjunction between additive and active material, for example, through chemical bonds or in situ co‐preparation (cf. Section 3).

Beside the active material and the conductive agent, composite electrodes usually contain binder materials. Binders ensure both the thorough blending of the active material and the conductive additive as well as a sufficient mechanical stability of the composite electrode.^21^ The most common binders are fluorinated polymers, such as poly(tetrafluoroethylene) (PTFE) and poly(vinylidene difluoride) (PVdF), which provide high electrochemical stability, binding capability, and electrolyte absorption ability.^22^ Carboxymethyl cellulose (CMC) is usually used if the composite is processed in an aqueous medium. Furthermore, CMC metal salts, poly(acrylate)s, or poly(3,4‐ethylenedioxythiophene)–poly(styrenesulfonate) (PEDOT:PSS) offer additional ions or semiconductivity, thus enhancing the performance of the composite electrode.^23^

## 3 Materials

### 3.1 Quinones

**Scheme 2:**
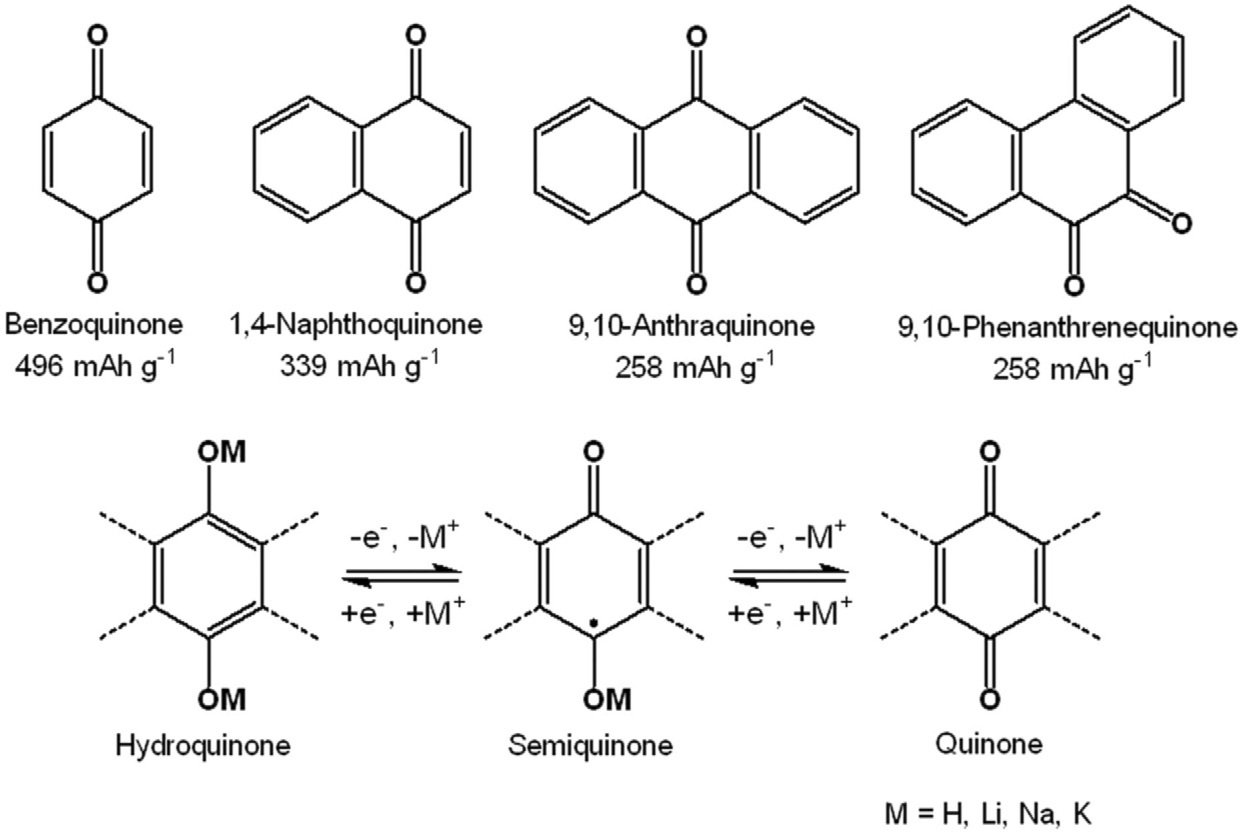
Top: Molecular structures and theoretical specific capacities of common quinones. Bottom: General redox mechanism of quinones.

Quinones represent the most popular group of organic active materials for electrochemical energy storage.^24^ They offer a stable and reversible redox chemistry, a wide range of electrochemical potentials, and a facile synthetic access.^25^ The electrochemical charge storage is based on the transition between the reduced hydroquinone and the oxidized quinone form (Scheme 2). Usually, quinones undergo two redox processes that are close in potential, but they can melt into one apparent two‐electron process when suitable functional groups and/or electrolyte systems are chosen. Thus, redox systems are possible that can store two electrons per molecular unit but possess a stable working voltage. During the redox transition, the quinone accepts (or releases) cations—in most cases either protons (in aqueous systems) or alkali metal ions (e.g., Li^+^, Na^+^, K^+^). Thus, quinones are often considered as organic metal‐insertion materials, as alternatives to inorganic insertion systems, such as LiFePO_4_.

Benzoquinone is the archetypical quinone, offering the simplest possible quinoid structure and, due to its comparably low mass, a high theoretical specific capacity of 496 mAh g^−1^ (for two electrons). Thus, several approaches have been undertaken to functionalize the basic molecule to optimize its properties with regard to battery application. Yao and co‐workers studied a benzoquinone with four sodium phenoxide groups in a hybrid sodium cell, which showed a sloping voltage (2.4 to 1.8 V) and 150 mAh g^−1^ but only lost 35 % of its capacity over 400 cycles.^26^ Similarly, a dimethoxybenzoquinone was synthesized and tested in a magnesium‐ion cell, showing a stable voltage and a capacity of 120 mAh g^−1^ but losing 80 % of capacity over only 30 cycles due to dissolution of the active material.^27^ Furthermore, a more elaborate 2,3‐dichloro‐5‐hydroxy‐6‐cyano‐1,4‐benzoquinone was tested against lithium but resulted in a voltage that ranged from 2 to 0.5 V.^28^ Matsubara et al. investigated a series of nine bisbenzoquinones with different halide and alkyl substituents.^29^ They achieved stable voltages of ca. 2.8 V in hybrid lithium cells with good capacities during the first cycles, but they suffered from substantial capacity losses already during the first 20 cycles. Sodium rhodizonate showed a four‐step voltage in a sodium‐ion cell but a good rate capability with the capacity at 50 C resembling 70 % of the 1 C‐value.^30^ However, the cell lost 20 % of its initial capacity over 100 cycles. The capacity loss could be reduced to 5 % by preparing nanorods of the active material.^31^ Park and co‐workers presented a triptycene‐like trisbenzoquinone with a two‐step voltage and a high initial capacity of 400 mAh g^−1^ in a hybrid lithium cell.^32^ Nevertheless, the cell lost 50 % over the first 20 cycles. Besides the small molecules, polymers containing benzoquinone units were investigated, in particular with regard to a low solubility of the active material in the electrolyte. However, they suffered from sloping voltages as well as low specific capacities.^33^

**Figure 2:**
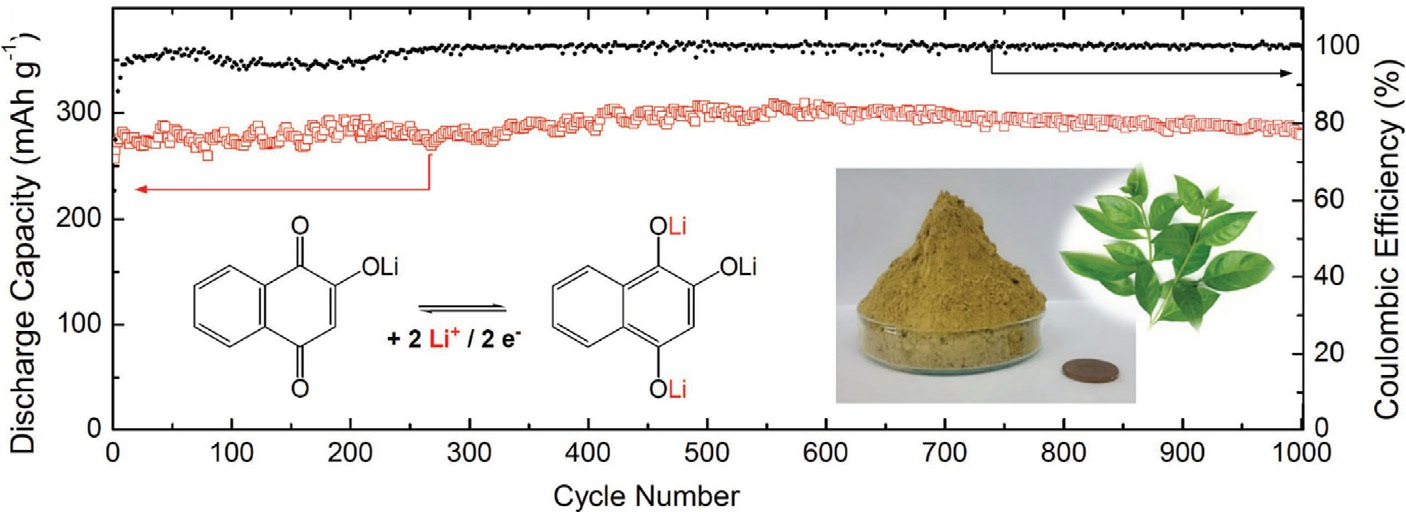
Long‐term performance of a hybrid lithium‐ion cell based on Lawsone (shown as a greenish powder in the right inset) as active material, revealing a discharge capacity that remains stable over 1000 consecutive cycles (reprinted with permission from Wiley, Copyright 2017).^34^

Naphthoquinones are less widespread compared to their smaller counterparts. Park et al. presented a hybrid lithium cell with 2‐hydroxy‐1,4‐naphthoquinone, called Lawsone, as active material, which featured a sloping voltage but a good specific capacity, which turned out to be stable over 1000 cycles (Figure 2).^34^ Naphtharazin, 5,8‐dihydroxy‐1,4‐naphthoquinone, and its chlorinated derivatives were likewise tested.^35^ Their lithium‐ion cells revealed high initial capacities of around 250 to 300 mAh g^−1^ but suffered from several voltage plateaus during the discharging as well as significant capacity fading during the first cycles. In contrast, a 2,3‐diamino‐functionalized naphthoquinone showed a stable discharge voltage as well as a high initial capacity and stability over 500 cycles.^36^ With regard to a decreased solubility, a polymerizable naphthoquinone was prepared via coupling to an azide‐bearing styrene, forming a naphthotriazolequinone, which was subsequently polymerized and used as active material in a lithium‐ion cell.^37^

**Figure 3:**
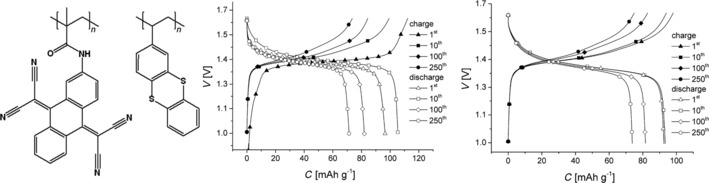
Left: Molecular structures of a TCAQ‐containing polymethacrylamide and poly(2‐vinylthianthrene), which constitute the active materials of a fully organic cell. Right: Respective charge/discharge curves at 1 C and 5 C, showing a capacity fade of 30 % over 250 cycles. (Reprinted with permission from Wiley, Copyright 2017).^43^

The anthraquinone motif in general offers high stabilities regarding both voltage and capacity and was extensively studied with regard to organic active battery materials in the past. Nevertheless, several derivatives were presented recently. Zhao et al. proved the applicability of a twofold sulfonated anthraquinone in a hybrid potassium cell.^38^ Emodin, a hydroxyl‐ and methyl‐functionalized species derived from plant extracts, allowed the construction of a cell with a stable voltage and a good initial capacity but showed a capacity loss of 70 % over 80 cycles.^39^ Using quinizarin (1,4‐dihydroxyanthraquinone) in combination with 2‐[1*H*‐indol‐2‐yl(1*H*‐indol‐3‐yl)methyl]phenol, a cell comprising two organic active materials (but still a Li^+^‐based electrolyte) was built but revealed a voltage curve that featured no distinct plateaus.^40^ Takeda et al. demonstrated an anthraquinone derivative in which carbon atoms in the aromatic rings were replaced by nitrogen atoms.^41^ A respective lithium‐ion cell showed a sloping voltage and an initial capacity of 250 mAh g^−1^, which decreased by 40 % within 30 cycles. The same contribution presented a cell based on tetracyano‐9,10‐anthraquinonedimethane (TCAQ), which was already introduced in 2014 as a redox‐active group in a polymeric active material.^42^ The cell incorporating the small TCAQ molecule featured a stable voltage and a good initial capacity, which, however, decreased to 40 % over 30 cycles.^41^ In another work, by Schubert and co‐workers, the polymeric TCAQ was used to build a fully organic cell with a thianthrene‐polymer‐based counter electrode, which featured a relatively high voltage of 1.4 V, a good capacity, and a reasonable capacity fade of 30 % over 250 cycles (Figure 3).^43^ Furthermore, a polymer based on 9,10‐di(1,3‐dithiol‐2‐ylidene)‐9,10‐dihydroanthracene (exTTF) was used in a hybrid zinc cell, resulting in a voltage of ca. 1.2 V and a capacity loss of only 5 % over 1000 cycles.^44^

**Figure 4:**
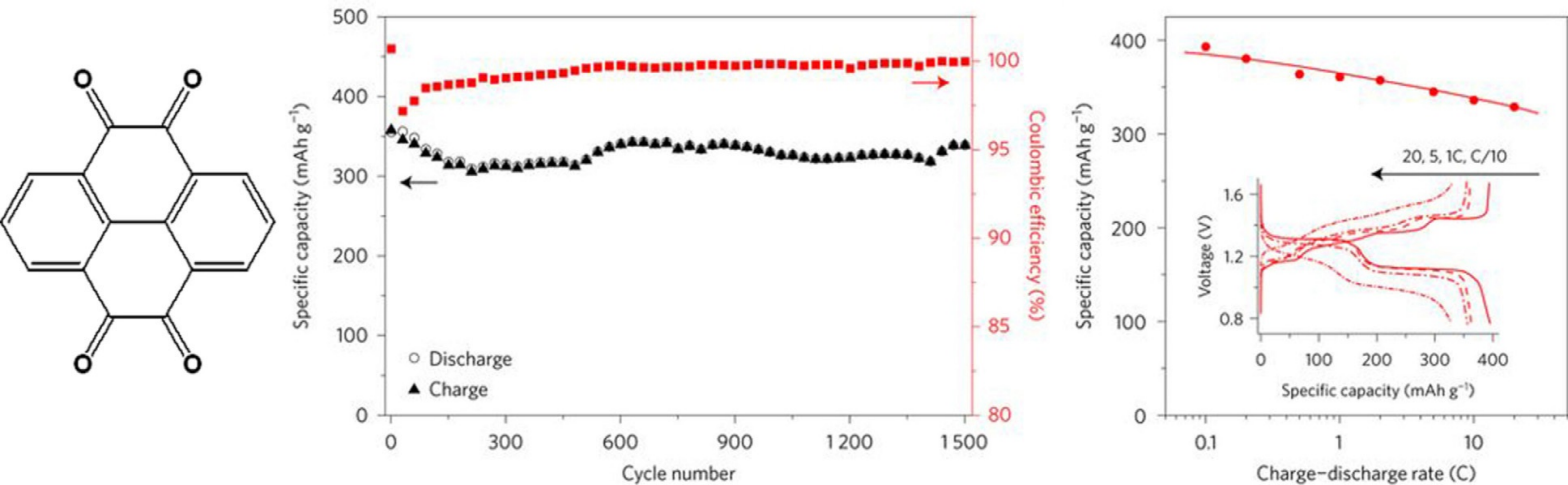
Left: Molecular structure of pyrene‐4,5,9,10‐tetraone. Right: Performance of a respective cell with a PbO_2_ counter electrode. The capacity remained nearly stable over 1500 consecutive charge/discharge cycles, while the voltage–capacity curves show a capacity loss of only 20 % by changing from a charge/discharge rate of 0.1 C to 20 C. (Reprinted with permission from Springer Nature, Copyright 2017).^47^

Further quinoid moieties are less common, but a few examples do exist. Diamino‐ and dinitrophenanthrenequinones were used in hybrid lithium cells, which revealed unstable discharge voltages and fast capacity fading.^45^ Likewise, a lithium‐ion cell comprising 5,7,12,14‐tetraaza‐6,13‐pentacenequinone as active material showed several discharge voltages and a capacity drop of 80 % over 250 cycles.^46^ Liang et al. presented a cell that used pyrene‐4,5,9,10‐tetraone and PbO_2_ as redox‐active electrode materials, which featured a high specific capacity of 350 mAh g^−1^, a capacity loss of only 5 % over 1500 cycles, and an excellent rate capability (Figure 4).^47^

**Figure 5:**
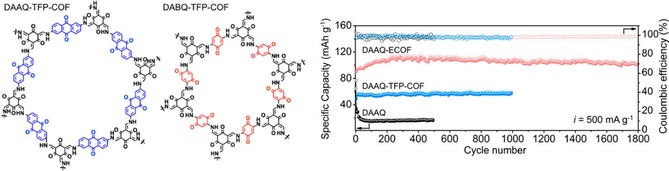
Left: Molecular structure of the anthra‐ and benzoquinone‐containing COFs (TFP=1,3,5‐tris(4‐formylphenyl)benzene). Right: Respective cycling data showing the high and stable capacity received for the exfoliated COF (ECOF) containing anthraquinone units (DAAQ‐ECOF) compared to the non‐exfoliated material (DAAQ‐TFP‐COF, with a likewise stable but lower capacity) and the unstable free 2,6‐diaminoanthraquinone (DAAQ). (Reprinted with permission from the American Chemical Society, Copyright 2017).50a

While the abovementioned polymers contain the active quinone moiety as side groups attached to the backbone, Liao and co‐worker presented polymers with a backbone formed by anthraquinone units, which are linked either via the 1‐ and 4‐ or 2‐ and 6‐carbon atoms and were utilized as active material in hybrid magnesium cells.^48^ Although both cells are comparable with regard to capacity and cycling stability, the former shows a stable discharge voltage, in contrast to a sloping voltage in the latter system. A polymer containing pyrene‐4,5,9,10‐tetraone in its backbone resulted in a sloping cell voltage and a capacity loss of 30 % over 100 cycles. Notably, the introduction of an ethynyl moiety into the backbone led to a significantly improved rate capability.^49^ Two‐dimensional covalent organic frameworks (COFs) containing redox‐active quinone moieties were presented by Wu et al.,50b Abruña et al.,50c and Wang et al.50a Applying exfoliated few‐layer nanosheets of the COFs in combination with a lithium counter electrode, a cell featuring a discharge voltage of 2.8 V and a capacity that is stable over 1000 cycles was demonstrated (Figure 5).50a

#### 3.1.1 Quinone‐sulfur polymers

**Figure 6:**
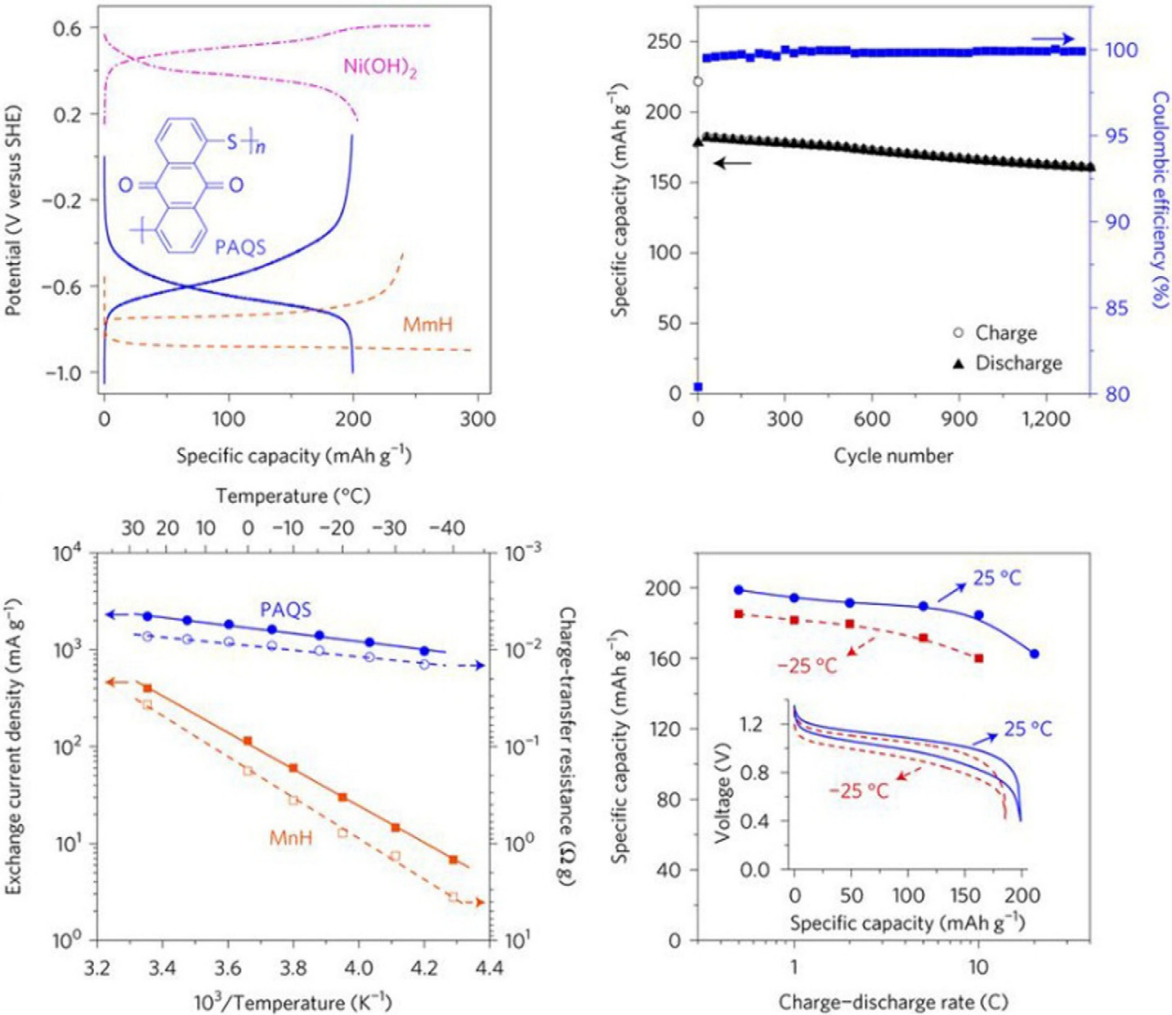
Performance of a PAQS‐Ni(OH)_2_ cell. Top left: Potential profiles of both electrodes during charging and discharging, compared to a metal–hydride electrode (MmH), showing stable, single plateaus. Top right: Course of the charge/discharge capacity over 1300 cycles with a loss of ca. 10 %. Bottom left: Exchange current density and charge‐transfer resistance of the polymer electrode and a metal–hydride electrode (MnH) within a temperature range of −40 to 30 °C, demonstrating the improved temperature independence of the polymer electrode. Bottom right: Specific capacity of the cell at different charge/discharge currents and temperatures, revealing only small losses of the capacity at higher currents and lower temperature. (Reprinted with permission from Springer Nature, Copyright 2017).^47^

Poly(quinonyl sulfide)s represent another popular group of quinone‐based redox‐active polymers. As for most polymeric systems used as active battery materials, they combine a low solubility with almost unaltered electrochemical characteristics. In the case of sulfide polymers, the sulfur linkers provide, depending on their length, potentially additional redox activity. While this can increase the provided capacity, such systems often suffer from sloping voltages or decreased stabilities.^51^ Yao and co‐workers presented a cell based on poly(anthraquinonyl sulfide) (PAQS) and Ni(OH)_2_ as active electrode materials and achieved a stable discharge voltage of 1.0 V and a capacity of 190 mAh g^−1^, which lost only 10 % over 1300 cycles. Furthermore, the obtained cell showed a noticeable temperature stability (Figure 6).^47^ A sulfide polymer derived from sodium‐phenoxide‐functionalized benzoquinone revealed a good performance in a hybrid sodium cell losing only 15 % capacity over 500 cycles.^52^

#### 3.1.2 Quinones linked to conductive substrates

**Scheme 3:**
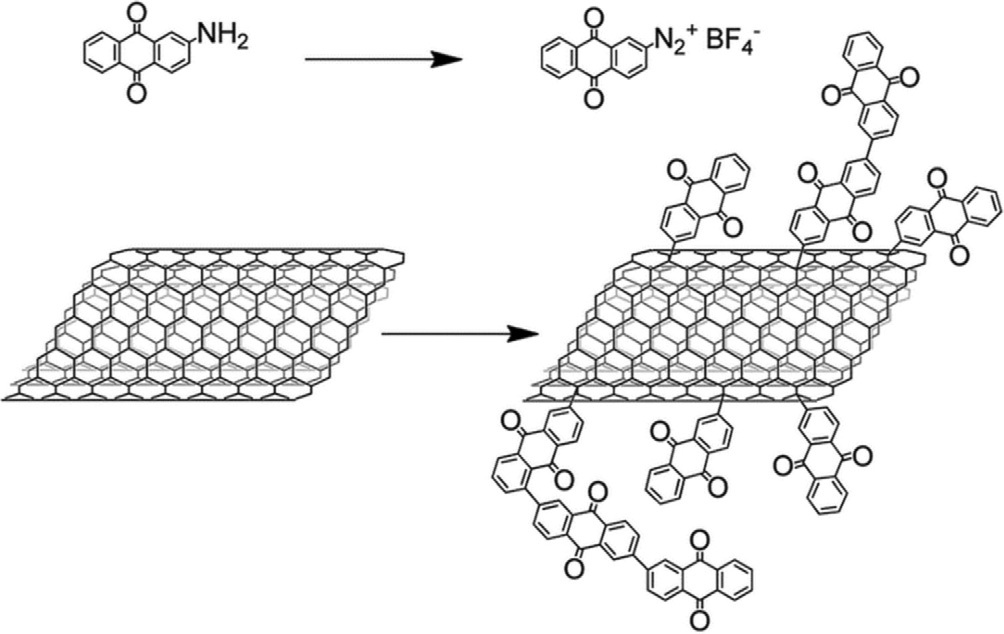
Modification of MWCNTs with anthraquinone (reprinted with permission of the Royal Society of Chemistry, Copyright 2016).^53^

As already stated, electrodes made of organic active materials usually contain significant amounts of conductive additive, mostly carbon materials, to ensure charge transport between the active material and the current collector. Consequently, the interaction between the organic compound and the carbon additive is crucial for the performance of the cell. Thus, several groups aim at the improvement of those interactions, for example, through the introduction of covalent bonds or exploitation of π–π interactions. Notably, the former approach is rather rare compared to the latter. Nevertheless, Campidelli et al. prepared multi‐walled carbon nanotubes (MWCNTs) with anthraquinone moieties that were covalently attached via reductive diazonium coupling (Scheme 3). Organic electrodes without conductive additives or binders were prepared and investigated in a hybrid lithium cell, resulting in a sloping voltage (2.2 to 1.7 V) and a capacity of 100 mAh g^−1^ that was stable at least for 50 cycles.^53^ The application of a reduced graphene oxide (RGO) with anthraquinone attached via azide coupling in a lithium‐ion cell resulted in a discharge curve without a distinct voltage plateau.^54^

Non‐covalent coupling of quinones and conductive carbon materials relies on the π–π interactions between the aromatic systems of the quinones and the graphene or carbon nanotubes. Those systems are usually prepared either by in situ synthesis of the redox‐active compounds in a carbon‐containing suspension or by co‐dispersion of both materials. Several demonstrated cells suffered from a working voltage that changed by more than 1 V during discharging,^55^ most likely due to a significant capacitive contribution of the large‐surface graphene materials in those composites or because of a poor cycling stability.^56^ In other cases, the intimate linkage of active material and conductive carbon led to an increased stability^57^ or rate capability.^58^ Nevertheless, for all of the abovementioned cells, additional conductive carbon additive was used for the preparation of the electrodes. In contrast, Wei et al. prepared poly(2,5‐dihydroxyl‐1,4‐benzoquinonyl sulfide) on single‐walled carbon nanotubes (SWCNTs), which was subsequently used as electrode material without further conductive additive or binder. The resulting hybrid lithium cell showed a capacity of 120 mAh g^−1^ with only 15 % loss over 500 cycles.^59^

**Figure 7:**
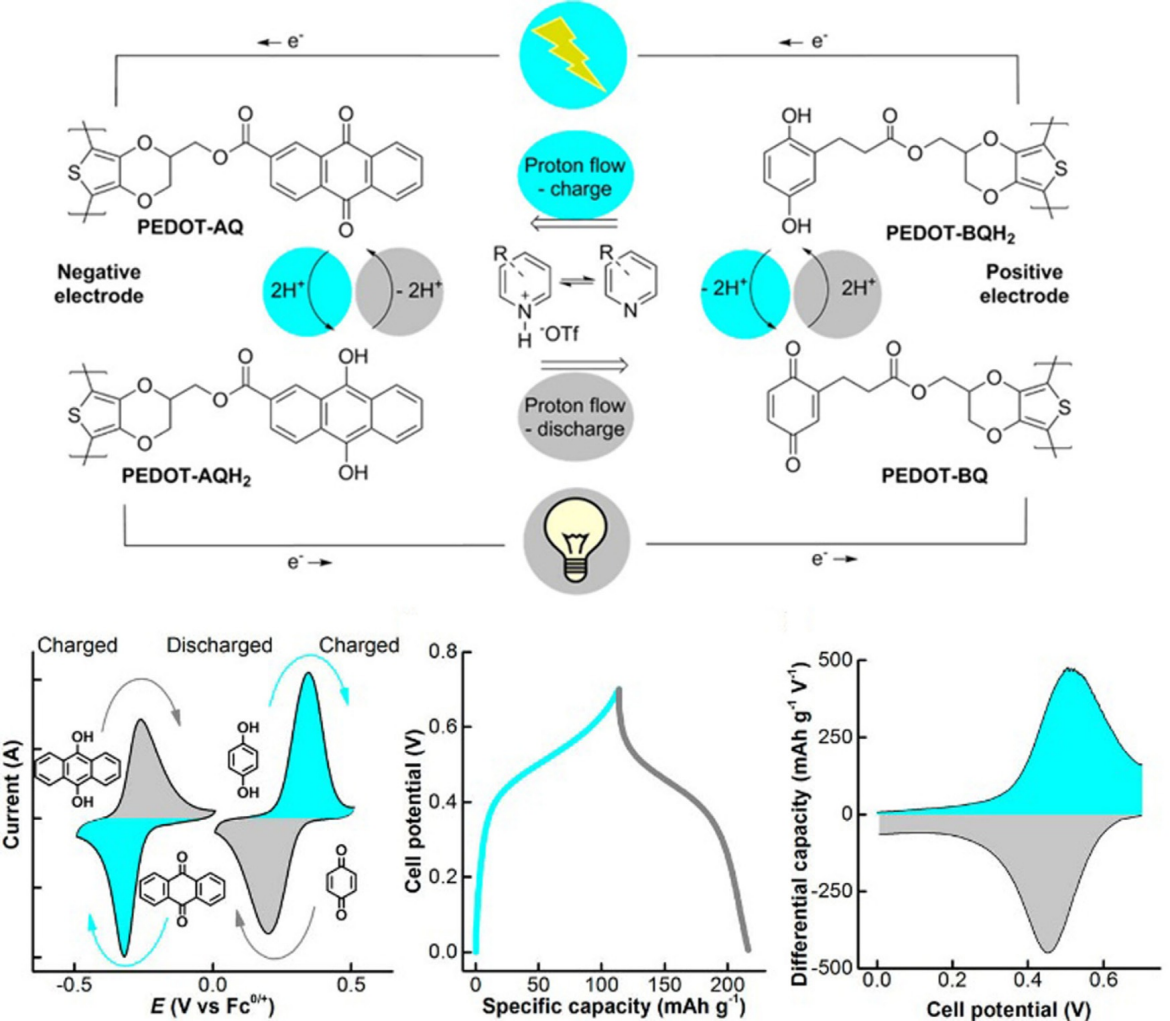
Top: Working principle of a poly(EDOT‐quinone)‐containing fully organic cell based on proton exchange via a pyridine electrolyte. Bottom: Related electrochemical characteristics, in particular the cyclovoltammograms of the active materials poly(EDOT‐anthraquinone) and poly(EDOT‐benzoquinone) (left), the charge/discharge voltage curve of a respective cell (middle), and a differential capacity plot derived from the latter data resembling the voltammetry experiment (right). (Reprinted with permission from the American Chemical Society, Copyright 2017).^62^

Beside carbon, other conductive materials were linked to the quinone active materials as well. Chen and co‐workers encapsulated benzoquinone into TiO_2_ spheres to decrease the solubility while retaining electrical conductivity.^60^ The resulting lithium‐ion cell showed a good initial capacity of 300 mAh g^−1^ but lost around 20 % during the first 100 cycles. Furthermore, several systems were proposed based on a conductive polymer with quinone side groups. However, most reports did not present a working cell.^61^ In contrast, Sjödin et al. showed an all‐organic cell, which used poly(EDOT–benzoquinone) and poly(EDOT–anthraquinone) as active electrode materials as well as a metal‐free, pyridine‐based electrolyte (Figure 7). However, the cell featured an unstable voltage and capacity.^62^

In summary, quinones still represent the most popular and promising class of organic active electrode materials in terms of specific capacity and stability, in particular when they are integrated in polymeric compounds. Thus, several all‐organic systems have been presented that are based partly or solely on quinone derivatives.

### 3.2 Diimides and dianhydrides

**Scheme 4:**
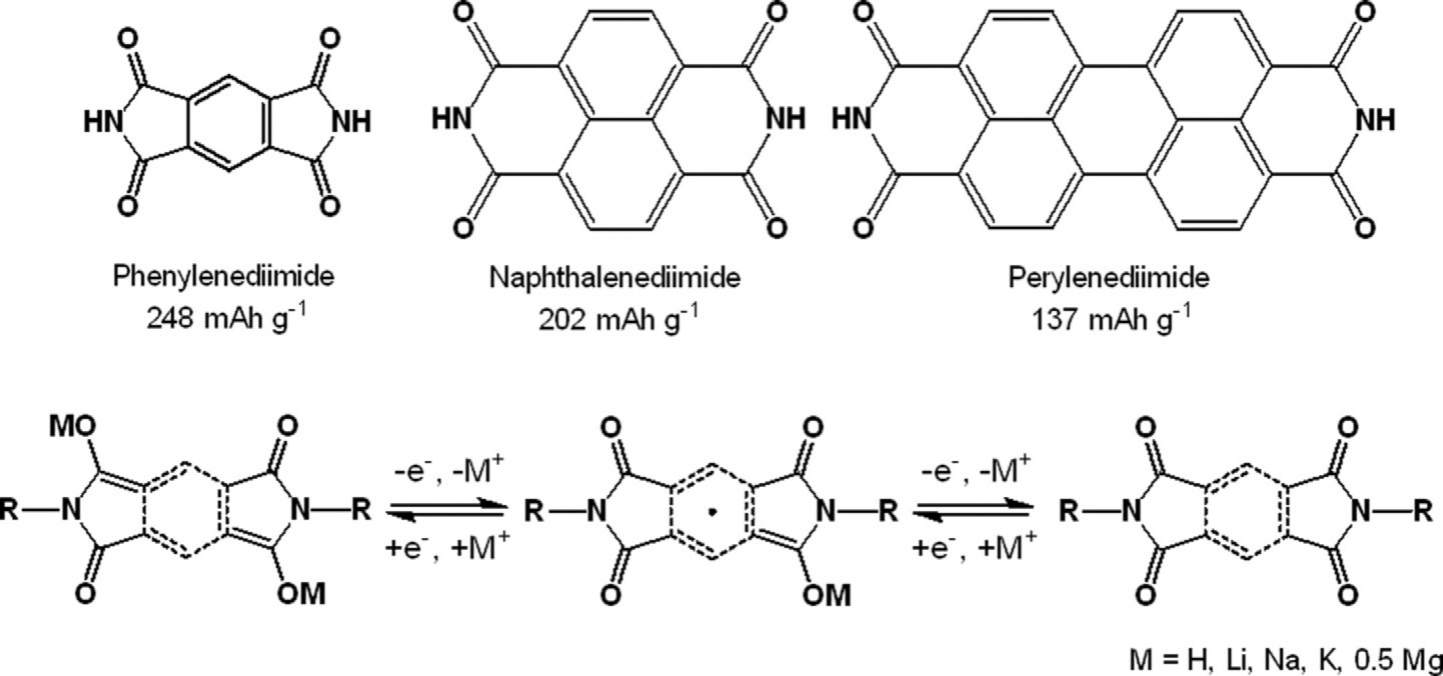
Top: Molecular structures of common aromatic diimides with theoretical specific capacities. Bottom: General redox mechanism of aromatic diimides.

Aromatic diimides offer a broad range of redox potentials, stable electrochemical processes, and a two‐electron‐storage capability per molecule.^63^ In addition, they tend to stack, leading to a low solubility in many electrolytes, thus improving the long‐term stability. Similar to quinones, the redox reactions are based on the transformation between two carbonyl and two hydroxyl groups, with the radical transition state being stabilized through the aromatic system, and the diimides can be applied as organic metal‐insertion materials (Scheme 4).

Phenylenediimide is the simplest aromatic diimide, and, although it is less electrochemically stable compared to its polycyclic counterparts according to theory,^64^ several derivatives were prepared for an application in organic batteries. Kothandaraman and co‐workers tested an acetate‐functionalized phenylenediimide in a lithium‐ as well as a sodium‐ion cell.^64^, ^65^ Although both cells showed a stable voltage plateau and comparable capacities, the latter revealed a significantly lower capacity retention. Rings composed of phenylenediimide and cyclohexyl moieties were likewise studied as active materials. Here, the performance of the cell depends strongly on the extent of the formed rings.^66^ In comparison to phenylenediimide, naphthalenediimide (NDI) possesses a larger aromatic system and a lower ring strain within the imide moieties. Thus, NDIs are usually more stable and more often used as active battery material. For example, cells with the NDI analogs of the abovementioned acetate‐containing diimides featured significantly improved long‐term stability.^64^, ^65^ In contrast, a likewise simple NDI, *N*,*N*′‐diphenyl‐NDI, showed a stable discharge voltage in a hybrid lithium‐ion cell but a capacity loss of 25 % over only 100 cycles.^67^ Perylene diimide features an even larger aromatic system, offering a potentially higher stabilization of charged states and was used by Cao and co‐workers in a hybrid lithium‐ion cell, which showed a stable voltage and a specific capacity of 110 mAh g^−1^ (80 % of the theoretical value) that remained stable over 200 cycles.^68^ An acetate‐functionalized derivative led to a lower capacity (which is expected due to the higher mass), which was stable over 100 cycles.^69^

**Figure 8:**
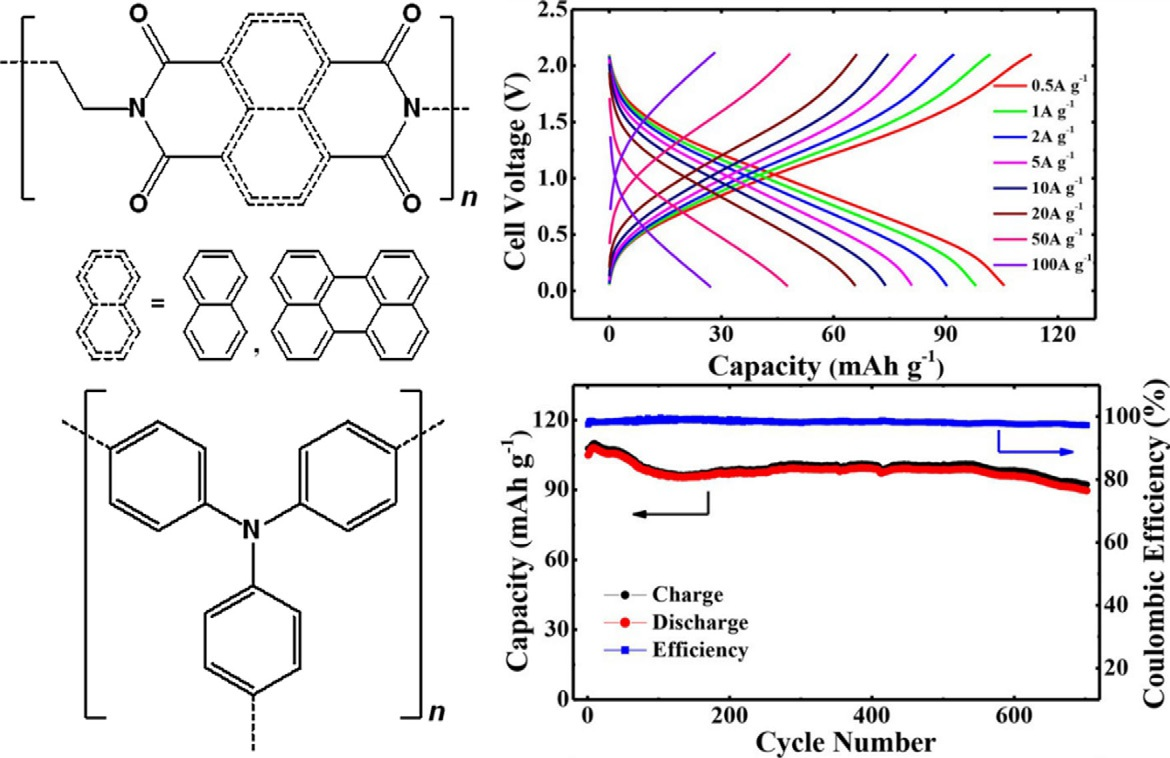
Left: Schematic representation of the active polymer materials of all‐organic poly(diimide)/poly(triphenylamine) cells. Right: Performance characteristics of a poly(NDI)/poly(triphenylamine) cell, revealing sloping charge/discharge voltages (top) but relatively stable capacities over 700 cycles (bottom). (Reprinted with permission from Wiley, Copyright 2017).^74^

**Figure 9:**
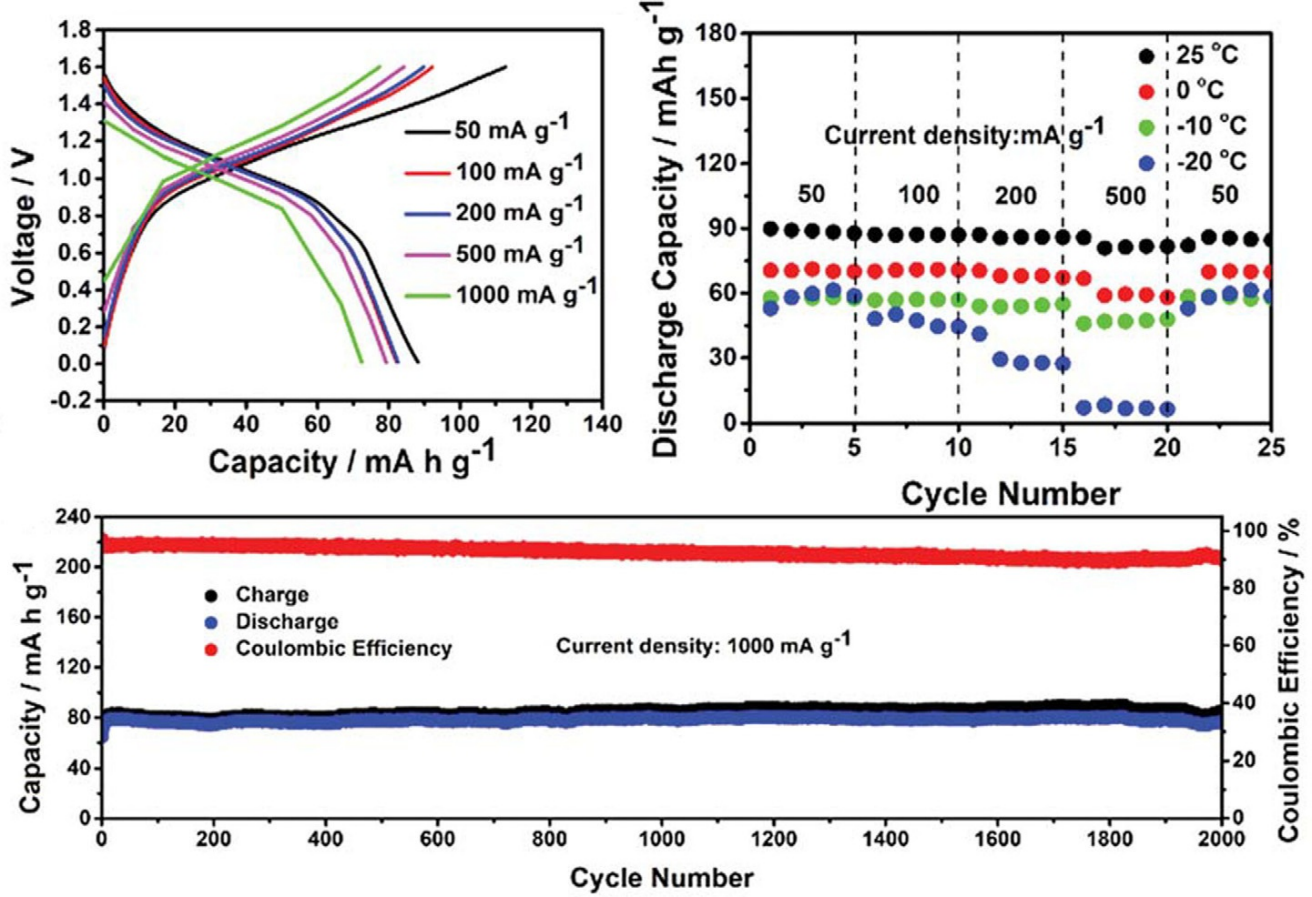
Performance of a poly(perylene diimide)/poly(triphenylamine) cell, in particular voltage profiles (top left) and capacities at different charge/discharge currents (top right), revealing a good rate capability of the system, as well as the capacity development over 2000 cycles (bottom), showing a good long‐term stability. (Reprinted with permission of the Royal Society of Chemistry, Copyright 2018).^74^, ^75^

To enhance the long‐term stability, also polymers containing NDI redox‐active moieties were thoroughly investigated. Dominko and co‐workers tested a simple polymer of nitrogen‐linked NDI units in a hybrid magnesium‐ion cell. Notably, the rather low initial capacity of 30 mAh g^−1^ increased over 100 cycles to 75 mAh g^−1^, which was explained by a swelling of the electrode.^70^ A phthalein and a tri(ethylene glycol) bridge were introduced into an NDI polymer by Mecerreyes and co‐workers.^71^ The resulting polymers were applied in lithium‐ion cells and showed capacities of 120 and 80 mAh g^−1^, respectively (corresponding to 82 and 75 % of the theoretical capacities), which remained stable over at least 100 cycles. Xu et al. incorporated a sulfonyl moiety as linking unit and, after the electrolyte was optimized, achieved a flat voltage plateau as well as a specific capacity of 120 mAh g^−1^ that remained stable over 400 cycles after a period of equilibration.^72^ Due to an additional redox‐active unit, the application of a carbonyl linker led to an even higher capacity of 160 mAh g^−1^, which was stable over at least 50 cycles.^73^ Dong et al. used an ethylene‐linked NDI polymer to build an all‐organic cell with a polytriphenylamine counter electrode and a highly concentrated (21 m) aqueous lithium bis(trifluoromethanesulfonyl)imide (LiTFSI) electrolyte.^74^ The resulting working voltage strongly decreased during the discharge, but the capacity loss was only 15 % over 700 cycles (Figure 8, right). Likewise, Lu et al. built a fully organic cell from an ethylene‐linked perylene diimide polymer and polytriphenylamine using Mg(ClO_4_)_2_ in the electrolyte.^75^ Again, the discharge voltage was slightly sloping with an average of 1.0 V, but the capacity of 80 mAh g^−1^ remained stable over remarkable 2000 cycles. In addition, the cell showed a good rate capability (Figure 9). Another all‐organic cell was presented by Picard and co‐workers, based on hexyl‐linked perylene diimide and perylene tetracarboxylate.^76^ The cell featured a slightly sloping voltage with an average of 1.0 V and an initial capacity of 80 mAh g^−1^, which decreased only by 20 % over 200 cycles.

Aiming at an improved lithium intercalation, two‐dimensional polymeric networks were prepared from perylene diimide and triptycene or tri‐β‐ketoenamine moieties. Due to the increased mass per redox‐active unit, the specific capacities are low, but, in particular for the former system, the capacities are rather stable.^77^

**Figure 10:**
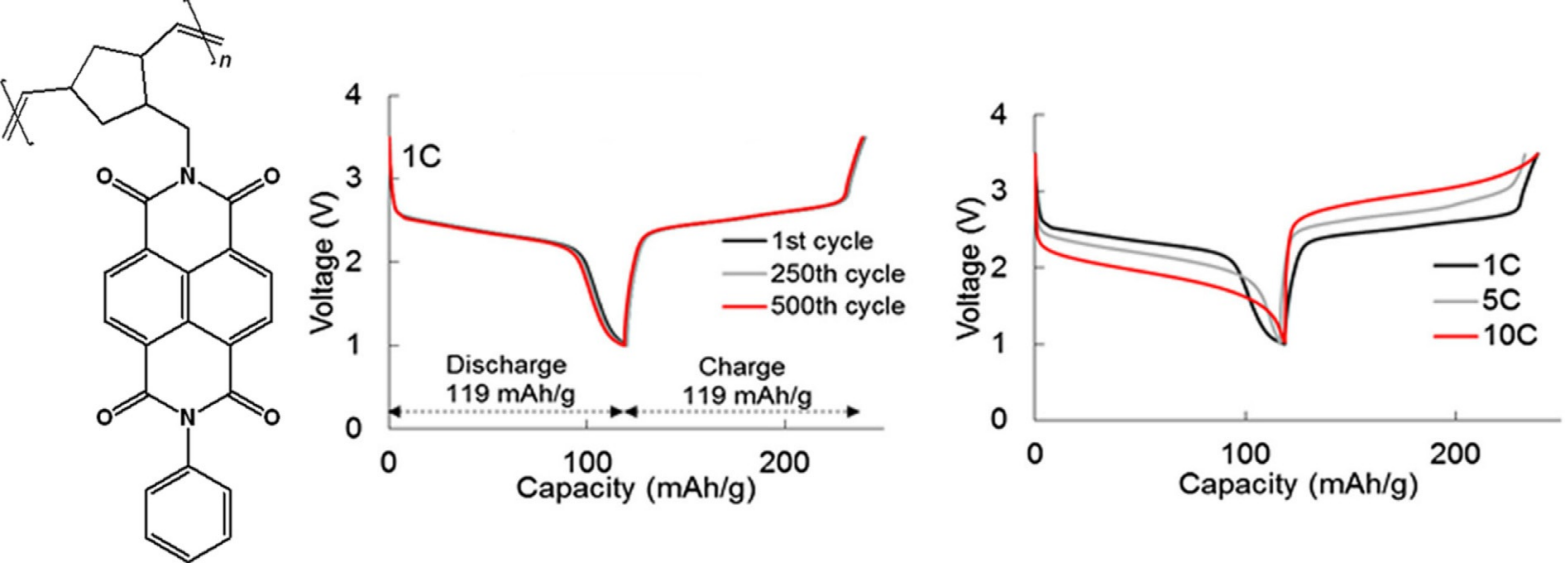
Left: Molecular structure of a norbornene‐NDI polymer used as active material in a hybrid lithium‐ion cell. Middle: Voltage profiles of the 1^st^, 250^th^, and 500^th^ charge/discharge cycles of the cell, revealing a stable performance. Right: Voltage profiles at different charge/discharge currents, demonstrating the good rate capability of the system. (Reprinted with permission the Materials Research Society, Copyright 2017).79a

Besides nitrogen‐connected polymer chains, also alternative configurations were studied. On the one hand, conjugated polymers from aromatic diimides linked via the carbocycles through phenylene or ethylene bridges were investigated but suffered from decreasing specific capacities.^78^ On the other hand, NDIs were introduced as side groups in polynorbornenes by Nishide and co‐workers.^79^ The resulting hybrid lithium‐ion cells showed stable voltages and capacities of 100 mAh g^−1^, which even remained stable over 500 cycles for a phenyl‐functionalized NDI (Figure 10).

#### 3.2.1 Mixed diimide‐quinone polymers

With the goal of achieving a higher specific capacity, several polymers containing an anthraquinone bridge linking the aromatic diimide moieties were prepared and tested in hybrid lithium‐ion cells.^80^ Since the resulting polymers are conjugated, they all showed a working voltage that decreased by ca. 0.5 V during discharge. According to the work of Xu et al., no significant differences between the 1,4‐ and 1,5‐linking anthraquinone are observable.80b For an NDI‐anthraquinone co‐polymer, a high specific capacity of up to 190 mAh g^−1^ was observed, which corresponds to 80 % of the theoretical value assuming four transferred electrons.80e

#### 3.2.2 Aromatic diimides linked to conductive carbon species

**Figure 11:**
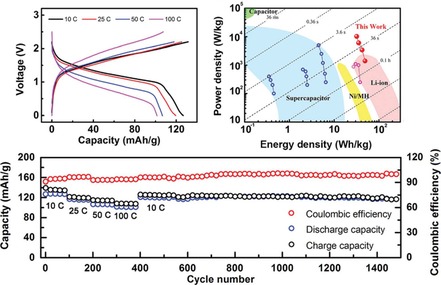
Characteristics of a fiber‐shaped hybrid lithium‐ion cell based on poly(NDI) on CNTs. Top left: Voltage curves at different charge/discharge rates demonstrating a good rate capability. Top right: Comparison of the performance of the cell with other systems via a Ragone plot. Bottom: Depiction of the charge/discharge capacity over 1500 cycles and several currents, showing a good long‐term stability and rate capability. (Reprinted with permission from the Royal Society of Chemistry, Copyright 2016).83d

Despite their conjugated nature, diimide‐based active materials, as most of the other organic active materials, are usually combined with electrically conductive materials, namely carbon materials, in organic battery electrodes to ensure sufficient charge transport.^12^ Aiming at a further enhancement of the charge transfer between the active material and the conductive carbon, π–π or ionic bonds between the diimides and graphene or carbon nanotubes (CNTs) are used to form composite compounds. Thus, alkylammonium‐functionalized naphthalene and perylene diimides were combined with RGO via co‐dispersion, exploiting ionic interactions between the cationic alkylammonium and anionic graphene oxide groups. Although the former showed a strongly decreasing voltage over the course of the discharge in a hybrid lithium‐ion cell,^81^ the perylene species resulted in a stable voltage and a capacity of 75 mAh g^−1^, which decreased by only 25 % over 500 cycles.^82^ Furthermore, composites of polymeric aromatic diimides and carbon materials were prepared, usually via in situ polymerization in a carbon‐containing dispersion. Similarly, poly(aromatic diimide)s on graphene and CNTs were synthesized.80d, 83 While most of the obtained hybrid cells showed sloping working voltages, high stabilities83b, 83d and improved rate capabilities80d, 83d were achieved due to the linkage to the conductive carbon compounds (Figure 11). Besides diimides, perylene dianhydrides were likewise studied in composites with conductive carbon materials for hybrid lithium‐ion cells.^84^ Here, the combination with RGO revealed the best performance with a stable voltage and a specific capacity of 130 mAh g^−1^, which even increased over the first 150 cycles by 15 %, as demonstrated by Cui et al.84d

Thus, aromatic imides are still among the most popular materials for organic battery electrodes. They allow the construction of cells that feature very stable capacities over several hundreds or even thousands of cycles.^75^, 79a, 83d Furthermore, they were successfully applied in all‐organic cells, in particular in combination with polytriphenylamines.^74^, ^75^, ^76^ The interesting approach of combining diimides with quinone units in co‐polymers showed, however, no significant improvement. In contrast, intimate linkage to conductive carbon species (mostly graphene and CNTs) enhanced both the long‐term cycling stability as well as the rate capability of the applied materials.80d, 83d

### 3.3 Other carbonyl compounds

#### 3.3.1 Terephthalates and other aromatic carboxylates

**Scheme 5:**
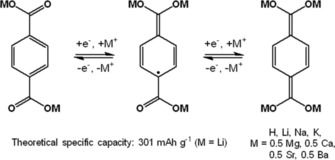
General redox mechanism of terephthalates.

The charge‐storage capability of terephthalates and other aromatic carboxylates is based on the reversible reduction of the carbonyl moieties of the carboxylate groups (Scheme 5). Like other carbonyl compounds, the terephthalates act as metal‐insertion compounds when they are used with a metal‐based electrolyte.^63^

Several functionalized terephthalates had been synthesized and were used in hybrid lithium‐ion cells. Most of them revealed significant capacity drops or showed no stable voltage at all.^85^ Nevertheless, a 2,5‐dimethyl‐terephthalate enabled a stable discharge voltage of 0.8 V against lithium and a stable specific capacity of 160 mAh g^−1^ over at least 50 cycles,^86^ whereas the application of a 2,5‐bis(phenylamine) terephthalate led to a voltage of 3.2 V but only a moderate capacity of 70 mAh g^−1^.^87^

**Figure 12:**
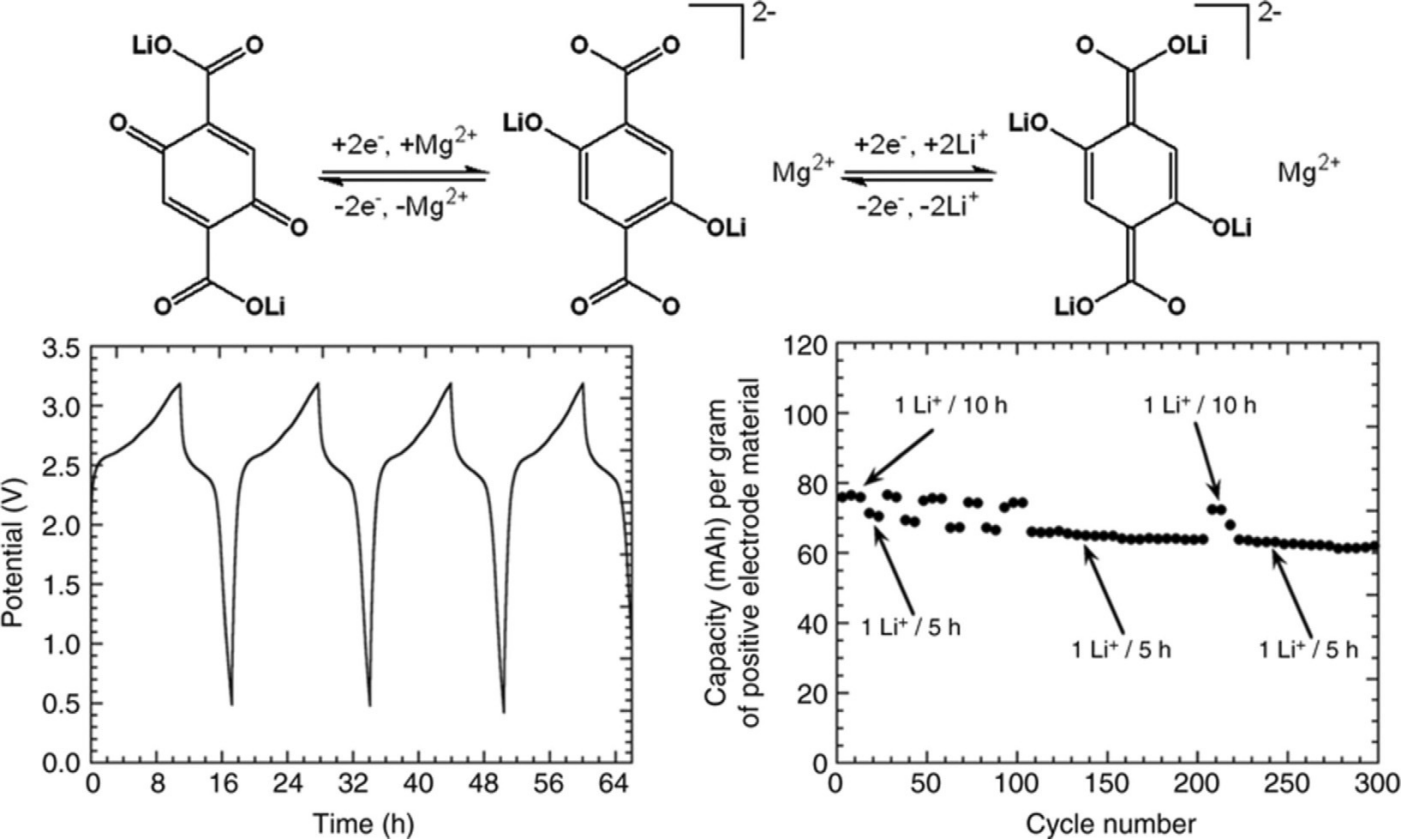
Top: Schematic representation of the mechanism of the two redox processes used for a symmetric all‐organic cell based on terephthalate active material and Li^+^‐containing electrolyte. Bottom left: Voltage profiles of the first four charge/discharge cycles of the cell. Bottom right: Capacity development of the cell over 300 cycles with a moderate loss of 20 %. (Reproduced under Creative Commons License, http://creativecommons.org/licenses/by/4.0/).^90^

Instead of lithium or other alkaline‐metal counter cations, Wang et al. used terephthalates with alkaline‐earth metal ions.^88^ Since they form different crystal structures with different ion pathways than their alkaline metal analogs, a better lithium‐ion transport through the electrode was anticipated. The studied hybrid lithium‐ion cells showed very high initial capacities of over 300 mAh g^−1^, which dropped to 170, 140, and 100 mAh g^−1^ during the first 10 cycles for calcium, barium, and strontium terephthalate, respectively (corresponding to 65, 80, and 50 % of the theoretical capacity), remaining constant over the next 40 cycles. A zinc terephthalate showed a similar behavior and reached a stable capacity of 180 mAh g^−1^ (75 % of theory) over 80 cycles after an equilibration of ca. 20 cycles in a hybrid lithium‐ion cell.^89^ Notably, the amorphous form revealed a much better performance compared to the crystalline ones. Poizot and co‐workers utilized a 2,5‐dihydroxyterephthalate with one magnesium and two lithium cations per molecule in a lithium‐organic cell, which featured a moderate capacity of 80 mAh g^−1^, remaining stable over at least 80 cycles.^90^ Subsequently, the authors applied the material in an all‐organic, Li^+^‐based, and symmetric cell, exploiting the carboxylate‐ as well as the quinone‐based redox processes of the compound (Figure 12). The observed capacity was 80 mAh g^−1^ and decreased by only 20 % over 300 cycles.

Polymers containing redox‐active terephthalate moieties were also tested as electrode materials, notably also poly(ethylene terephthalate) from recycled bottles,^91^ but the resulting cells mostly suffered from vastly decreasing discharge voltages.33b, 91, 92

#### 3.3.2 Terephthalates linked to conductive substrates

**Figure 13:**
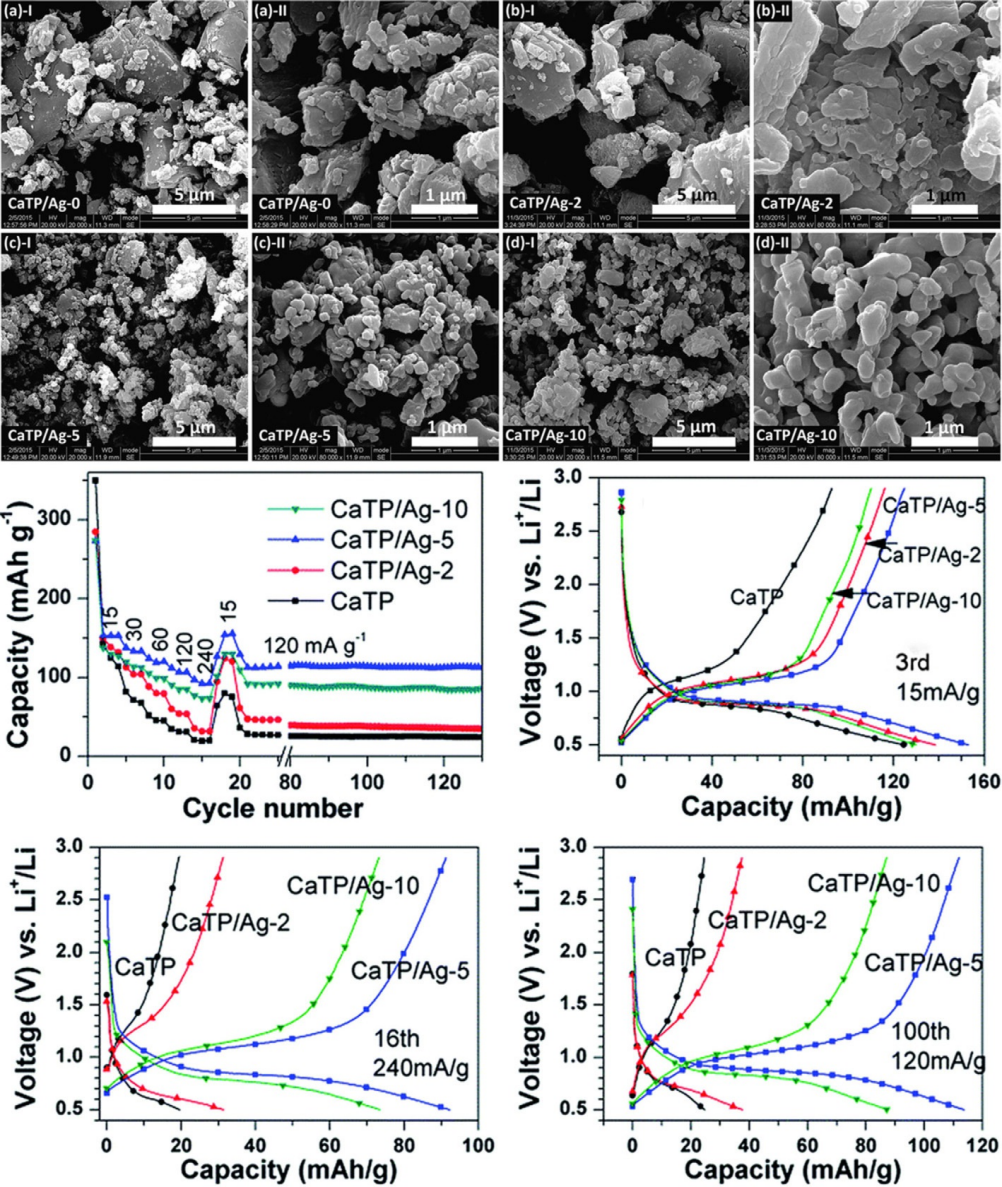
Top: SEM images of calcium terephthalate silver composites, showing a decreasing particle size and increasing particle uniformity with increasing silver precursor content. Bottom: Performance characteristics of respective hybrid lithium cells, in particular the charge/discharge capacity over 130 consecutive cycles and voltage profiles at different currents and silver precursor contents, demonstrating an optimum of the latter at 5 wt %. (Reprinted with permission from the Royal Society of Chemistry, Copyright 2016).^96^

As most organic materials that are used for electrochemical energy storage, terephthalates do not provide electrical conductivity that is high enough to ensure sufficient charge transport during charging and discharging. Thus, a conductive additive has to be added. Aiming at an optimum interaction, several composites in which the redox‐active and conductive material are closely connected were studied. The most common systems are terephthalates combined with graphene. A composite of sodium terephthalate and graphene, achieved via co‐dispersion, resulted in a hybrid sodium cell with a working voltage of only 0.3 V but a good initial capacity of 220 mAh g^−1^, which decreased by only 20 % over 500 cycles.^93^ In contrast, a comparable system based on potassium resulted in a sloping voltage of 1.2 to 0.8 V and an initial capacity of only 120 mAh g^−1^.^94^ In a mixed approach, which used potassium terephthalate on graphene in a sodium‐ion cell, also a decreasing discharge voltage and a specific capacity of 150 mAh g^−1^ but an improved rate capability compared to the graphene‐free cell were achieved.^95^ An alternative to the utilization of carbon as conductor is represented by the application of silver particles. Li and co‐worker prepared a composite of calcium terephthalate and silver via dispersion of the organic compound in a silver nitrate solution with subsequent drying and reduction of the silver ions.^96^ The built hybrid lithium‐ion test cell revealed a stable voltage of 1.0 V and a capacity of 90 mAh g^−1^. Notably, the silver content determined the rate capability, with an optimum at 5 wt % (Figure 13). The same group demonstrated a cell where the silver is formed in situ through the utilization of silver terephthalate. Within in the first cell cycle, the silver cations were irreversibly reduced and formed uniformly dispersed silver nanoparticles.^97^

With its low molar mass accompanied by a potential two‐electron storage ability, terephthalates offer the possibility of high specific capacities. Indeed, many systems with high initial capacities were demonstrated, but most of them suffered from significant losses during the first 100 cycles. Furthermore, compared to quinones and diimides, relatively low voltages were observed for respective hybrid metal cells. As for quinones and aromatic diimides, composites that possess a close connection between active and conductive materials were presented, which showed good stabilities and rate capabilities. However, they are clearly inferior to the best comparable quinone‐ or diimide‐based systems.

#### 3.3.3 Other aromatic carboxylates

**Figure 14:**
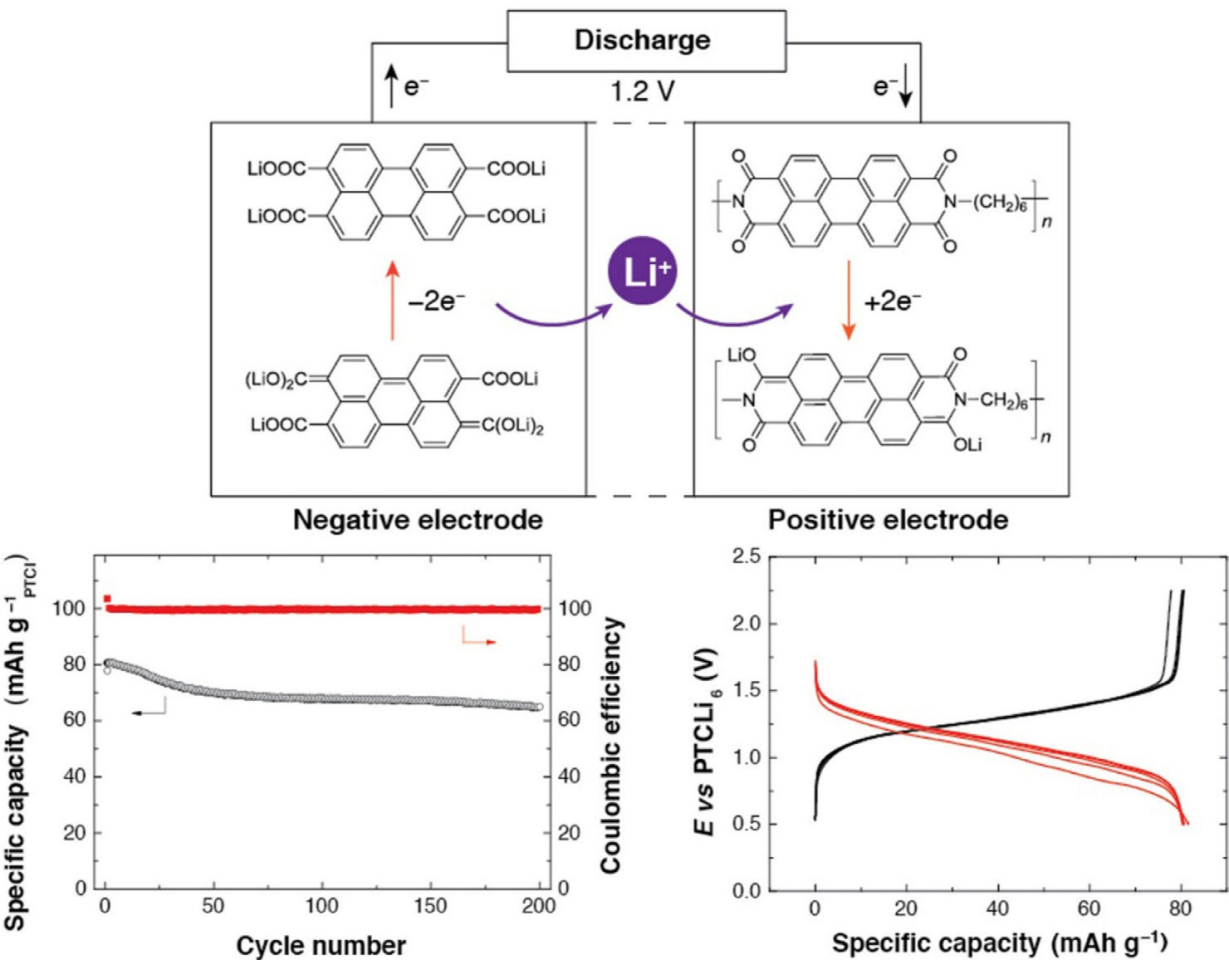
Top: Working principle of an all‐organic cell based on perylene‐tetracarboxylate and a poly(perylene‐diimide) as active materials and a Li^+^‐containing electrolyte. Bottom left: Capacity development of the cell over 200 charge/discharge cycles, revealing a drop of 20 % within the first 50 cycles followed by a stable performance over 150 cycles. Bottom right: Voltage curves of the first five cycles. (Reprinted with permission from the American Chemical Society, Copyright 2017).^76^

Derived from the original terephthalate motif, several other aromatic carboxylates were studied as active electrode materials, from biphenyl species over polycyclic aromatic compounds to heterocycles. Ramanujam and co‐workers compared 1,1′‐biphenyl‐4,4′‐dicarboxylate to benzil‐4,4′‐dicarboxylate and 1,1′‐biphenyl‐3,3′,4,4′‐tetracarboxylate. They found that the former lost its complete recharging ability within 50 cycles, while the benzil species preserved 80 % of its initial capacity of 160 mAh g^−1^.^98^ The tetracarboxylate showed an only moderate initial capacity of 110 mAh g^−1^, losing further 35 % over the first 100 cycles.^99^ 1,1′‐Biphenyl‐4,4′‐dicarboxylate and 4,4′‐*E*‐stilbenedicarboxylate were deposited on graphene, and the resulting materials were tested in hybrid potassium‐ion cells. Both systems showed low and sloping voltages, and the initial capacities were halved during the first 10 to 30 cycles.^100^ Furthermore, naphthalene‐2,6‐dicarboxylate was used in a hybrid lithium‐ and a hybrid sodium‐ion cell, which revealed working voltages of 0.8 and 0.6 V, losing 45 and 25 % of its initial capacity over the first 100 cycles.^101^ Naphthalene tetracarboxylate did not even show a voltage plateau in the first place.^102^ In contrast, Iordache et al. demonstrated a hybrid lithium‐ion cell using perylene‐3,4,9,10‐tetracarboxylate as active electrode material, which featured a stable voltage of 1.2 V and an initial capacity of 120 mAh g^−1^, losing 25 % over the first 200 cycles.^103^ Consequently, the material was also used in a fully organic cell, using a hexyl‐linked perylene diimide polymer as second electrode material.^76^ The cell possessed a slightly decreasing discharge voltage with an average at 1.1 V and a capacity of 75 mAh g^−1^, which showed a reasonable drop to 80 % within the first 200 cycles (Figure 14). However, further extension of the structure to benzoperylene hexacarboxylate resulted in a cell that lacked any recognizable voltage plateaus during discharging.^104^ The heterocyclic 2,5‐pyridinedicarboxylate was tested against sodium and potassium. In the former case, a two‐step voltage was observed, which was accompanied by a high initial capacity of 280 mAh g^−1^.^105^ The potassium‐based cell revealed a stable voltage but a lower capacity of 180 mAh g^−1^.^106^ However, both systems lost one fourth of their capacity during 100 cycles.

### 3.4 Stable organic radicals

**Scheme 6:**
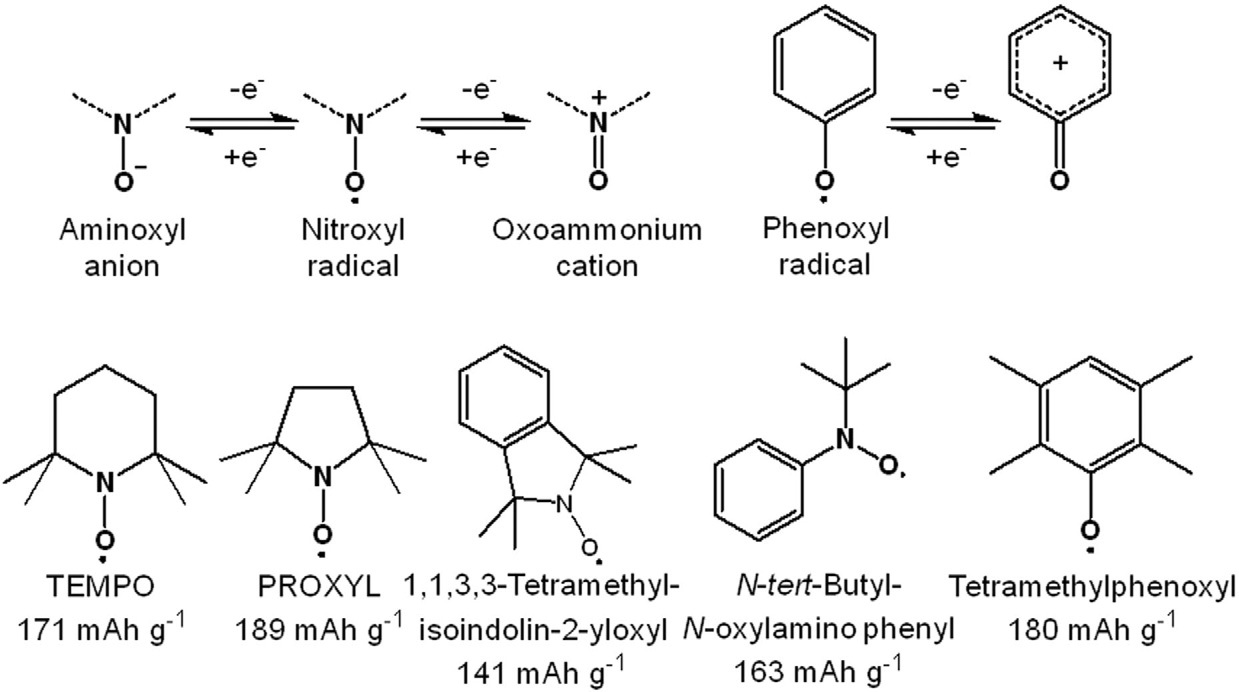
Top: Molecular structures of organic radical moieties and their preferential redox reactions. Bottom: Organic radicals recently used in organic cells.

Stable organic radicals are characterized by the presence of an unpaired electron within the molecule. They are stabilized through the introduction of sterically demanding substituents and electron resonance, thus impeding problems that are related to radical compounds, such as dimerization through the formation of bonds between radicals.11d, 107 In charge‐storage applications, organic radicals usually store one charge per redox‐active unit, leading to a non‐radical, thus more stable species in the charged state. Additionally, the redox processes of organic radicals are based only on the transfer of a single electron without larger structural changes of the molecule, which results in improved electron‐transfer rates.^13^ On the other hand, counterions have to migrate during redox processes to maintain electroneutrality, which affects the related redox kinetics. This can occur either via ion expulsion or uptake, depending on the ion types present and ion concentrations as well as the applied charge‐transfer rate, as recently shown for polymers containing TEMPO radicals.^108^ Nevertheless, high charging and discharging currents are possible leading to high power densities. The most common organic radical motifs are the nitroxyl and the phenoxyl radicals (Scheme 6).

#### 3.4.1 TEMPO‐based materials

**Figure 15:**
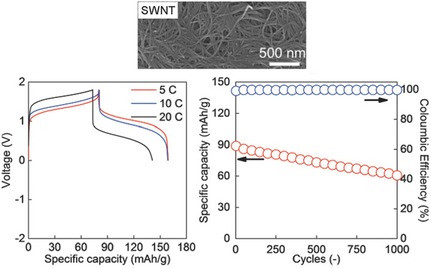
Top: SEM image of single‐walled CNTs prepared by means of eDIPS. Bottom: Performance characteristics of an all‐organic beaker‐type cell containing an anthraquinone‐based anode and a cathode from poly(TEMPO acrylamide) and single‐walled CNTs, in particular the voltage profiles at different charge/discharge currents (left) and the course of the capacity over 1000 consecutive cycles, revealing a loss of 30 % (right). (Reprinted with permission from Wiley, Copyright 2018).^113^

The TEMPO radical is the by far most popular organic radical and has been used in batteries since 2002.^10^ Thus, it is well known and most of the studies that were recently conducted focused on the variation and optimization of established systems. To enhance charge‐transfer rates through enhanced electronic conductivities, TEMPO‐containing polymers were combined with conductive carbon materials (graphite, graphene, CNTs, etc.) either by in situ polymerization on the material,^109^ by co‐dispersion,^110^ by encapsulation,^111^ or by grafting‐on approaches.^112^ The resulting materials, on the one hand, often showed a sloping or multistep voltage,^109^, ^110^, ^111^ most likely due to an additional, capacitive contribution by the high‐surface‐area carbon material. On the other hand, a significantly improved rate capability compared to simple composites of active material and conductive additive was achieved^111^, ^112^ and also a working cell without additives was demonstrated.^109^ Nishide and co‐workers used SWCNTs that were prepared by enhanced direct‐injection pyrolytic synthesis (eDIPS), which showed a high crystallinity and enabled the preparation of an electrode based on poly(2,2,6,6‐tetramethylpiperidinyloxy‐4‐yl acrylamide) (PTAm) with less than 5 % CNTs.^113^ The resulting electrode was applied in an all‐organic cell with poly(anthraquinone‐substituted ethylene imine) (PAQE) as counter electrode, using an aqueous sodium chloride electrolyte. The cell delivered a stable discharge voltage of 1.0 V, a remarkable rate capability, and an initial specific capacity of 80 mAh g^−1^, which retained 70 % after 1000 cycles (Figure 15). Alternatively, TEMPO units were coupled to a conjugated polymer, namely poly(dithieno[3,2‐b:2′,3′‐d]pyrrole, for enhanced charge transfer.^114^ Electrodes without additives were prepared via electropolymerization, leading only to a small mass loading of 0.1 mg cm^−2^. The resulting hybrid lithium‐ion cell revealed a decreasing discharge voltage around 3.3 V and an initial capacity of 60 mAh g^−1^.

**Figure 16:**
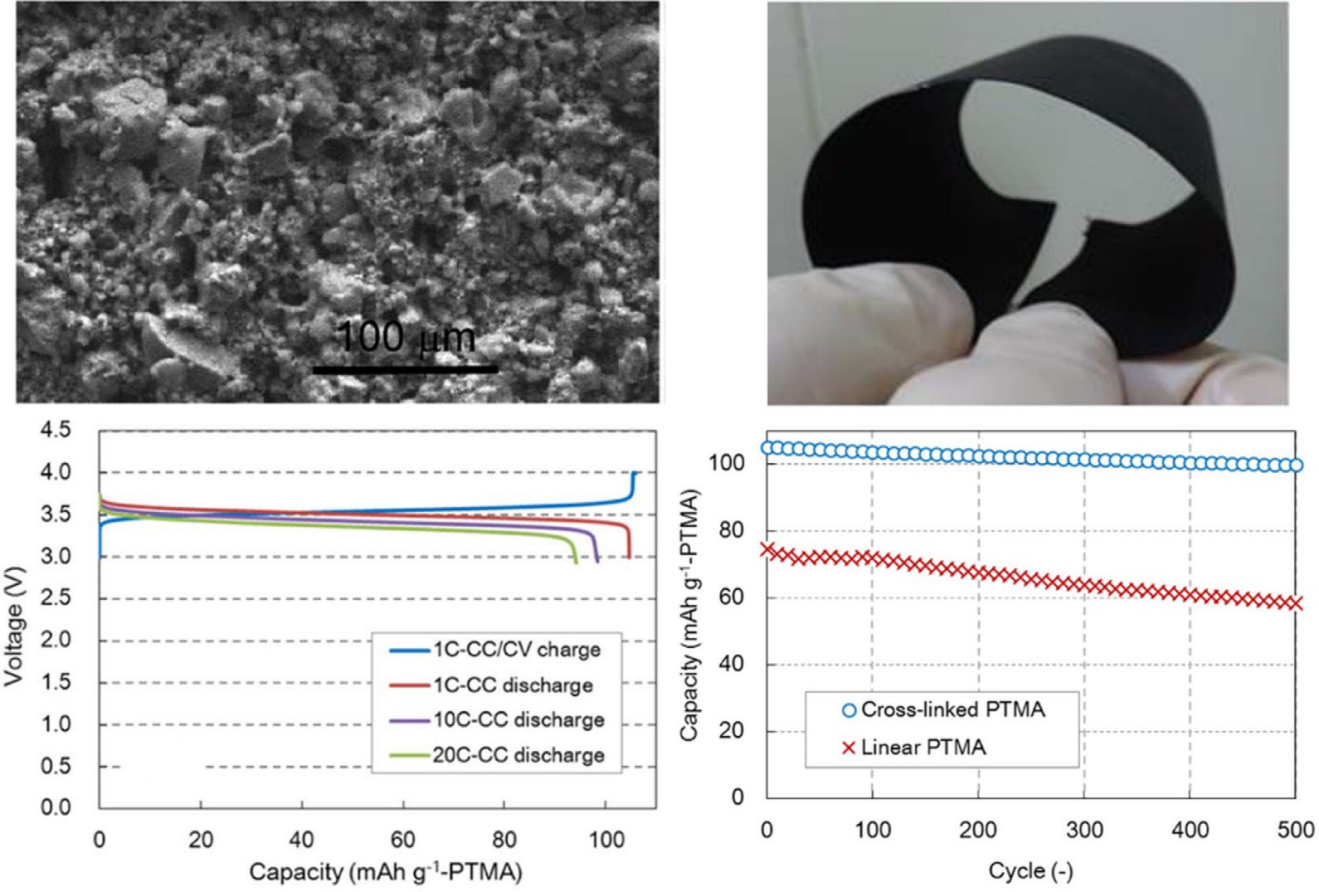
An electrode based on cross‐linked TEMPO gel polymer. Top left: SEM image of the electrode consisting of agglomerated particles. Top right: Photograph of the bent electrode. Bottom left: Voltage curves for the charging/discharging of a respective hybrid lithium‐ion cell at different currents. Bottom right: Capacity development over 500 cycles in comparison to a cell containing the non‐cross‐linked counterpart. (Reprinted with permission of the Electrochemical Society, Copyright 2017).^118^

Alternatively, systems with TEMPO units linked to conjugated polymer chains were studied in lithium‐ion cells, but while a polyaniline‐based material revealed a strongly sloping discharge voltage (between 3.7 and 2.8 V) and unstable capacities,^115^ a PEDOT‐containing polymer led to a very low initial capacity of 35 mAh g^−1^.^116^ With regard to an improved ionic conductivity, an imidazolium‐containing polymeric ionic liquid bearing TEMPO groups was synthesized, but a test cell did not show any distinct voltage plateau.^117^ For the same purpose, a COF with TEMPO units was prepared.50a However, the resulting lithium‐ion cell revealed a two‐step discharge voltage and, compared to its quinone counterparts (cf. Section 3.1), a low specific capacity.50a In contrast, Iwasa et al. presented a bendable hybrid lithium‐ion cell using a gel of a cross‐linked TEMPO polymer.^118^ The utilization of the gel electrode led to a stable voltage of 3.5 V, a capacity of 105 mAh g^−1^, which remained nearly stable over 500 cycles, as well as an excellent rate capability (Figure 16).

Beside the conductive additive, organic electrodes usually require a polymeric binder for mechanical stability and thorough contact among the components. Zhang and co‐workers developed a TEMPO‐containing polymer based on poly(ethylene maleic anhydride), which they applied in a binder‐free electrode.^119^ The related hybrid lithium‐ion cell showed a stable voltage of 3.6 V and a capacity of 80 mAh g^−1^, which decreased by only 10 % over remarkable 2000 cycles.

#### 3.4.2 Other stable organic radicals

Most other organic radicals that were studied for battery applications are, like the TEMPO itself, nitroxyl radicals. A didehydro‐PROXYL (2,2,5,5‐tetramethyl‐1‐pyrrolidinyloxy) radical was introduced in a triphenylamine polymer.^120^ The obtained hybrid lithium‐ion cell revealed a prominent voltage plateau at 3.7 V but also a second one at 2.7 V, which was assigned to the polytriphenylamine. The overall capacity was 120 mAh g^−1^, losing 10 % over 100 cycles. Subsequently, the same group attempted to enhance the electrochemical performance by the introduction of MWCNTs, but no significant improvement was achieved.^121^ A polymer of the 1,1,3,3‐tetramethylisoindolin‐2‐yloxyl radical showed a comparable performance in a lithium‐ion cell,^122^ whereas a polyphosphazene bearing an *N‐tert*‐butyl‐*N*‐oxylamino phenyl radical unit resulted in a higher initial capacity of 145 mAh g^−1^ but a capacity drop of 35 % during the first 50 cycles.^123^ A phenoxyl radical was used in a polynorbornene in form of tetramethylphenoxyl units, but a built hybrid lithium‐ion cell showed a capacity of only 60 mAh g^−1^, losing 20 % capacity over the first 100 cycles.^124^

In conclusion, no new organic radicals that represent promising candidates for organic batteries were revealed recently. Nevertheless, systems based on already established motifs, namely on TEMPO and didehydro‐PROXYL, were further optimized, in particular with regard to higher electrical and ionic conductivities.

### 3.5 Conjugated polymers

**Figure 17:**
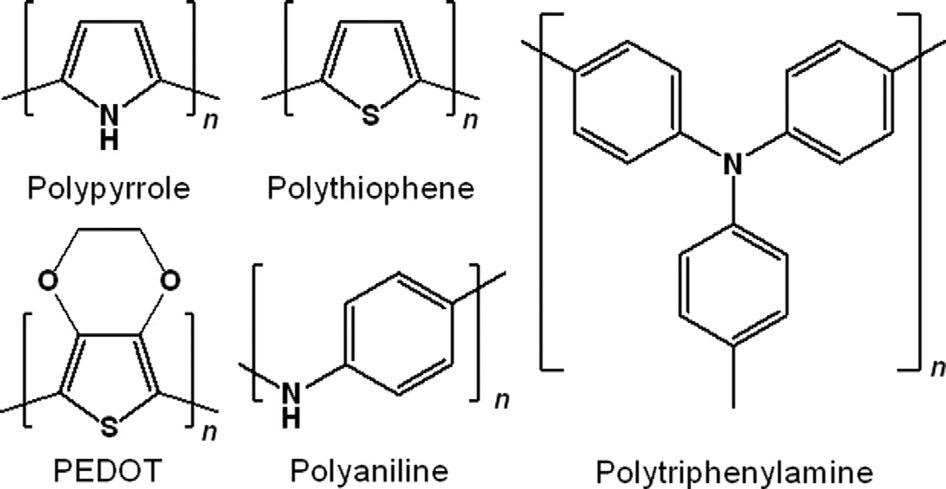
Molecular structures of conjugated polymers that were tested in organic batteries.

The first organic compounds that were used for electrochemical energy storage belonged to the class of conjugated polymers.^8^ However, since those systems were not able to provide stable voltages and capacities, the first approaches were quickly discarded. Indeed, a main drawback of conjugated polymers is the fact that their redox potentials usually depend on their state of charge, leading to the described sloping working voltage.^125^ This is caused by the semiconductor‐like electronic band structure of conjugated polymers, which is formed by the overlap of the π orbitals of the single “monomer” units of the polymer.^126^ Nevertheless, the very same band structure provides conjugated polymers with electronic and ionic conductivities that are superior to most other redox‐active organic compounds when they are partly oxidized (or reduced), that is, doped. Thus, a decreased need for conductive additives can be achieved, making conjugated polymers interesting candidates for organic batteries (Figure 17). Furthermore, most conjugated polymers can be obtained via electrochemical polymerization, enabling an in situ formation in electrode setups.

#### 3.5.1 Polypyrrole, polythiophene, and polyaniline

Polypyrrole, polythiophene, polyaniline, and their derivatives (e.g., PEDOT) belong to the most common conjugated polymers and are applied in various optical, electronic, and electrooptical devices due to their advantageous redox and optical properties. Thus, they were also tested as active battery materials, namely polypyrrole,^127^ polythiophene,^128^ PEDOT,^129^ poly(dithieno[3,2‐b:2′,3′‐d]pyrrole),^114^ and polyaniline.^115^, ^130^ However, all the presented systems showed significantly sloping discharge voltages or strongly decreasing capacities, which makes them unsuitable for battery application.

#### 3.5.2 Polytriphenylamine

**Figure 18:**
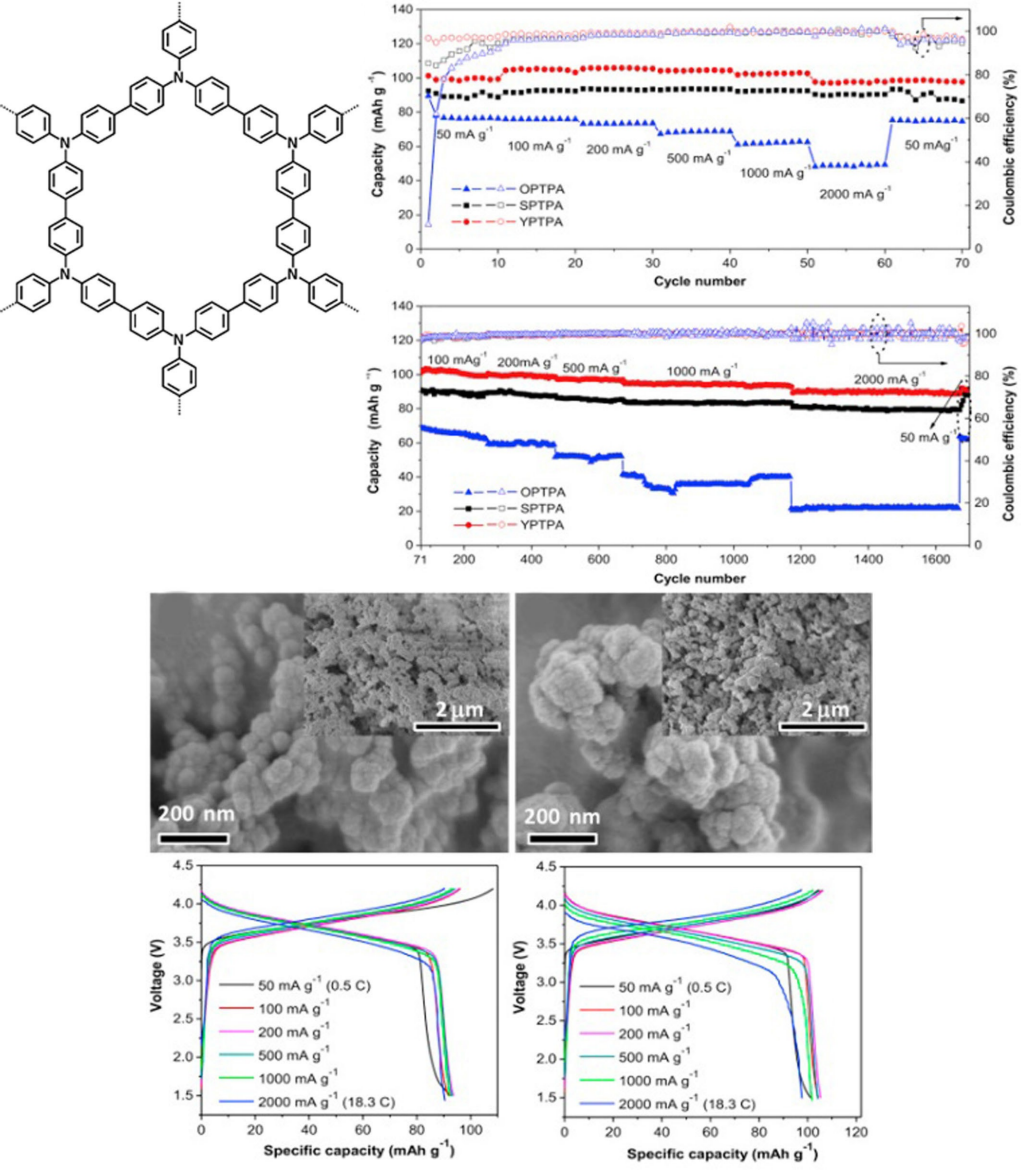
Two‐dimensional polytriphenylamine networks in hybrid lithium cells. Top left: Molecular structure. Top right: Capacity development at different charge/discharge currents and over 1600 cycles for both systems (black and red), demonstrating both good rate capabilities and long‐term stabilities. Middle: SEM images of the obtained microporous structures with different specific surface areas, showing aggregates of 60–80 nm in both cases. Bottom: Charge/discharge curves of the respective hybrid lithium cells at different currents. (Reprinted with permission from Elsevier, Copyright 2016).^131^

Polytriphenylamines differ from the abovementioned polymers in their ability to form two‐dimensional structures. Jiang and co‐workers prepared polytriphenylamine networks that form microporous structures, which give rise to an enhanced ion transport (Figure 18).^131^ The resulting hybrid lithium‐ion cell revealed a stable discharge voltage, a capacity of 100 mAh g^−1^, which remained stable over 200 cycles, and a good rate capability.

Through replacing some of the triphenylamine units by triphenylbenzene moieties, Zhang and co‐workers created a two‐dimensional network with different pore sizes. The resulting hybrid lithium‐ion cell showed a performance that resembled mostly that of a polytriphenylamine‐based analog, except for a slightly improved rate capability.^132^ A cell based on a polytriphenylamine and a graphite electrode and a potassium hexafluorophosphate‐based electrolyte was demonstrated, displaying a stable voltage but a low capacity of 60 mAh g^−1^ and a coulombic efficiency of only 80 %.^133^ Dong and co‐workers prepared a polytriphenylamine on MWCNTs via in situ polymerization of triphenylamine in a dispersion of MWCNTs.^121^ Subsequently, the authors built a hybrid lithium‐ion cell without additional conductive additives, which revealed a stable voltage of 3.7 V and a capacity of 100 mAh g^−1^, retaining 85 % after 100 cycles.

Further electrode materials based on functionalized polytriphenylamines were prepared. A polymer from triphenylamine and aniline, a triphenylamine–phenylene–imine polymer, and a methoxy‐bearing polytriphenylamine suffered from strongly changing discharge voltages.^134^ In contrast, lithium‐ion cells containing polytriphenylamines with cyano and methyl groups revealed stable voltages and capacities as well as good rate capabilities.134c

Conjugated polymers are still popular active materials for organic batteries. However, most of the studied compounds revealed unstable discharge voltages, as long known for such systems, as well as low and decreasing capacities. As an exception, polytriphenylamine proved to be a promising basis for organic electrodes, with several examples that provided stable voltages and capacities as well as excellent rate capabilities. Thus, polytriphenylamines were even already used in all‐organic cells combined with aromatic diimides (cf. Section 3.2).^74^, ^75^

### 3.6 Aromatic compounds

#### 3.6.1 Heterocycles

**Figure 19:**
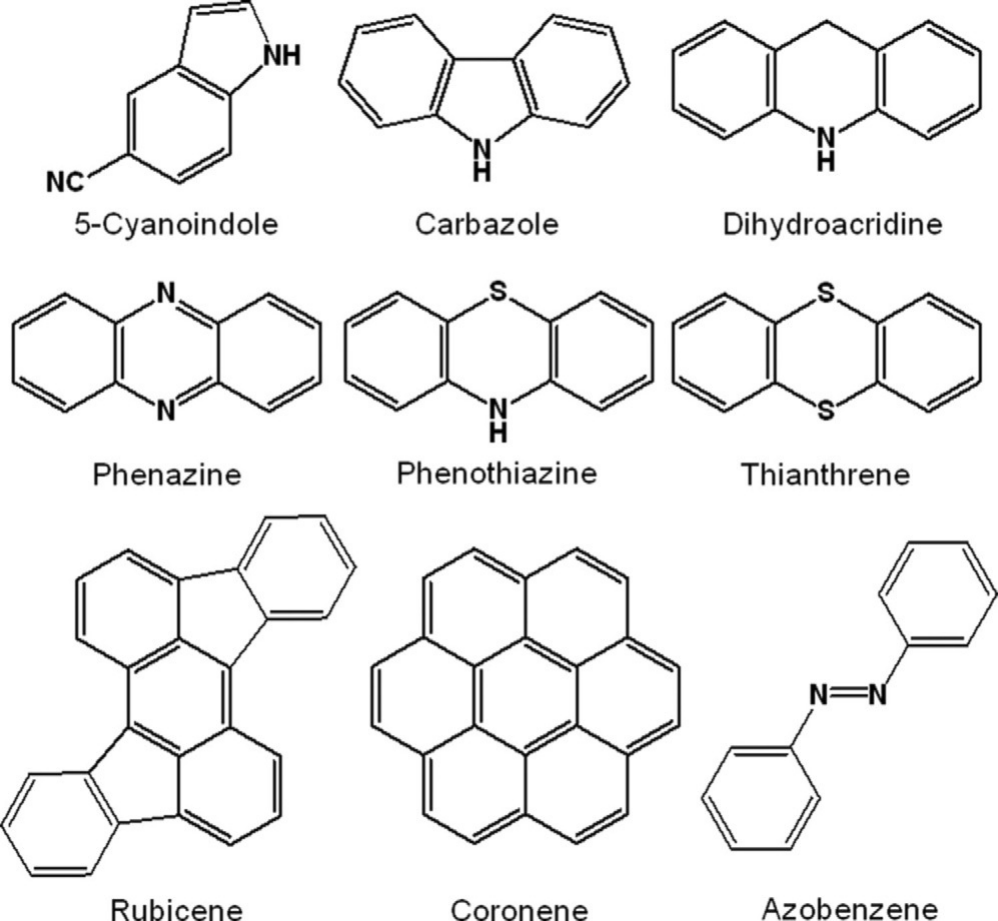
Heterocyclic moieties and other aromatic compounds that were tested as active materials for organic batteries.

Several heterocycles have been tested as organic charge‐storage materials in batteries (Figure 19). Their aromatic nature enables them, in principle, to reversibly accept or release electrons. Cai et al. built a hybrid sodium‐ion cell using poly(5‐cyanoindole). The cell showed a decreasing discharge voltage, but its capacity, which reached 100 mAh g^−1^ after a short equilibration, decreased only by 15 % over 1000 cycles.^135^ Higashibayashi and co‐workers prepared electrodes based on dicarbazole and di(dihydroacridine) for lithium‐ion cells.^136^ Notably, the applicability of the dimers depended strongly on the linkage. While an only C−C‐coupled carbazole dimer revealed no usable working voltage, its counterpart that was C−C‐ and *N*−*N*‐coupled revealed a slightly sloping voltage around 3.5 V and a capacity of 50 mAh g^−1^, which remained stable over 20 cycles. A likewise assembled dihydroacridine dimer resulted in a stable voltage and a slightly higher initial capacity of 80 mAh g^−1^ but lost 35 % over only 20 cycles. Further promising heterocycles were presented, in particular phenazine,^137^ phenothiazine,^138^ and thianthrene,^43^ with the latter being even applied in a working, fully organic cell (Figure 3).

#### 3.6.2 Other aromatic systems

Some larger polycyclic aromatic compounds were also investigated with regard to an application in organic batteries (Figure 19). 5,12‐Diaminorubicene was used in a hybrid lithium‐ion cell, resulting in a sloping voltage and a capacity loss of 35 % over the first 60 cycles.^139^ Coronene, on the other hand, delivered a stable voltage of 4.0 V and a capacity that was rather low (35 mAh g^−1^) but retained 85 % after 950 cycles.^139^ Furthermore, Wang and co‐workers prepared a series of azobenzenes and tested them as active materials in hybrid sodium‐ion cells.^140^ Notably, the cells were the more stable the more carboxylate groups were attached to the azobenzene, resulting in a capacity of 170 mAh g^−1^, retaining 95 % after 100 cycles.

## 4 Concluding Remarks

During the recent years, many efforts were made with respect to the development of active organic electrode materials for electrochemical energy storage. Several new structural motifs were studied regarding their electrochemical behavior and their applicability in batteries. However, up to now, the most successful systems are the already established ones, in particular simple aromatic quinones (benzo‐, naphtho‐, and anthraquinone), aromatic diimides (naphthalene and perylene diimide), and the stable organic radical TEMPO (cf. Sections 3.1, 3.2, and 3.4.1, respectively). A large amount of research was dedicated to the optimization of these compounds by the introduction of functional groups, integration into polymers, or combination of different redox‐active moieties. In particular, an increasing interest in the development of mixed materials containing a redox‐active organic species and an electrically conductive compound (e.g., graphene, carbon nanotubes (CNTs), as well as silver particles) is apparent. The components are either connected directly to each other via chemical bonds (e.g., using clicking techniques) and π–π interactions (in particular for graphene and CNTs), or conductive nanoparticles are formed in situ within the active‐material network. The driving force for this research is the desire for making the use of conductive additives as well as binders redundant in future organic electrodes, thus increasing the overall specific capacity of the battery. Beside the established redox units, other aromatic materials, namely terephthalates, poly(triphenylamine)s, and some heterocyclic compounds (cf. Sections 3.3.1, 3.5, and 3.6.1, respectively) proved to be promising candidates. Consequently, the number of fully organic approaches is increasing, and several working all‐organic cells were presented (Table 2) during the last years, revealing relatively high voltages,^43^ capacities,^90^ and stabilities.^75^

**Table 2:** Recently demonstrated all‐organic cells.

Positive pole	Negative pole	*V* [V]	*C*_spec_ [mAh g^−1^] (stability)	Remarks	Ref.
poly(thianthrene)	poly(TCAQ)	1.4	100 (−30 %/ 250 cycles)	–	43
poly(EDOT‐benzoquinone)	poly(EDOT‐anthraquinone)	0.5	80 (−15 %/ 100 cycles)	metal‐free electrolyte	62
poly(triphenylamine)	poly(NDI‐ethylene)	1.5–0.5	90 (−15 %/ 100 cycles)	aq. 21 m LiTFSI electrolyte	74
poly(triphenylamine)	poly(perylene diimide‐ethylene)	1.4–0.9	80 (stable over 2000 cycles)	Mg(ClO_4_)_2_; good rate capability	75
poly(perylene diimide‐hexylene)	perylene‐3,4,9,10‐tetracarboxylate	1.1	75 (−20 %/ 200 cycles)	–	76
MgLi_2_(2,5‐dihydroxy‐terephthalate)	MgLi_2_(2,5‐dihydroxy‐terephthalate)	0.8	140 (−25 %/ 20 cycles)	symmetrical	90
poly(TEMPO acrylamide)	poly(ethylene imine‐anthraquinone)	1.0	80 (−30 %/ 1000 cycles)	aq. NaCl electrolyte	113

There is still necessity for improvement and optimization but also many possibilities to do so due to the large toolbox that is provided by organic chemistry. However, not only the active materials offer many possibilities of development, but also the accompanying components, namely conductive additives as well as electrolyte salts and solvents, allow a comprehensive optimization with regard to enhanced interactions and synergies among the different parts. Hence, although there are already promising approaches and results at present, there is still much work to be done on the way to commercial everyday all‐organic batteries.

## Conflict of interest

*The authors declare no conflict of interest*.

## Biographical Information

Christian Friebe was born in Erfurt (Germany) and studied chemistry at the Friedrich Schiller University Jena (Germany), where he graduated in organometallic chemistry in 2008. He conducted his PhD studies on the electrochemical and spectroscopic characterization of transition‐metal complexes at the Friedrich Schiller University Jena under the supervision of Prof. U. S. Schubert and received his PhD in 2013. He currently works as a postdoctoral fellow in the group of Prof. U. S. Schubert in the field of energy‐storage materials.

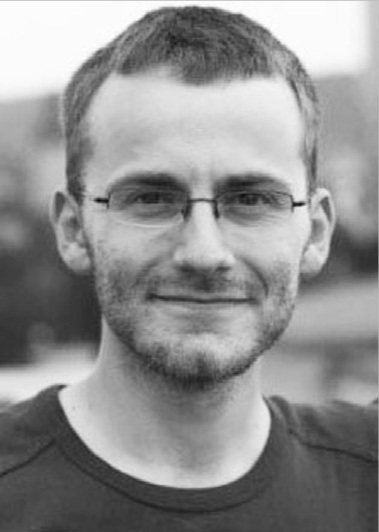

## Biographical Information

Alexandra Lex‐Balducci was born in Fürstenfeld (Austria). She studied technical chemistry at Graz University of Technology (Austria), where she obtained her PhD at the Institute of Chemistry and Technology of Organic Materials in 2008. Afterwards, she joined the group of Prof. M. Winter at the University of Münster (Germany) for a postdoctoral fellowship. From 2009 till 2015, she was the scientific leader of an independent junior research group at the MEET Battery Research Center at the University of Münster (Germany). In 2015, she joined the group of Prof. S. Passerini at the Helmholtz Institute Ulm (HIU)/ Karlsruhe Institute of Technology (KIT) (Germany). Since 2016, she works as a senior researcher in the group of Prof. U. S. Schubert at the University of Jena (Germany) in the field of organic batteries with the focus on polymer electrolytes for organic batteries.

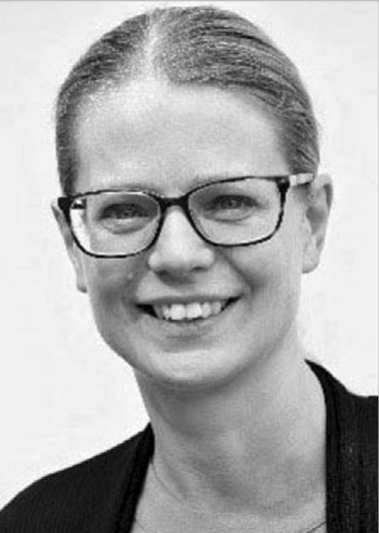

## Biographical Information

Ulrich S. Schubert was born in Tübingen (Germany) in 1969. He studied chemistry at the Universities of Frankfurt and Bayreuth (both Germany) and the Virginia Commonwealth University, Richmond (USA). His PhD work was performed under the supervision of Prof. C. D. Eisenbach (Bayreuth, Germany) and Prof. G. R. Newkome (Florida, USA). After a postdoctoral training with Prof. J.‐M. Lehn at the Université Strasbourg (France), he moved to the Munich University of Technology (Germany) to obtain his habilitation in 1999 (with Prof. O. Nuyken). From 1999 to spring 2000, he held a temporary position as a professor at the Center for NanoScience (CeNS) at the LMU Munich (Germany). From June 2000 to March 2007, he was Full‐Professor at the Eindhoven University of Technology (Chair for Macromolecular Chemistry and Nanoscience), the Netherlands. Since April 2007, he is Full‐Professor at the Friedrich Schiller University Jena (Chair of Organic and Macromolecular Chemistry), Germany and Founding Director of the Center for Energy and Environmental Chemistry Jena (CEEC Jena).